# Photo-driven transient frustrated Lewis pairs for catalytic hydrogenation

**DOI:** 10.1039/d6sc02736a

**Published:** 2026-06-25

**Authors:** Jin Lin, Shuanghui Chen, Kang-Shun Peng, Yung-Hsi Hsu, Shuchun Li, Longji Cui, Hansong Zhang, Yongjie Wang, Xue Feng Lu, Sibo Wang, Kunlong Liu, Sung-Fu Hung, Xinchen Wang

**Affiliations:** a State Key Laboratory of Chemistry for NBC Hazards Protection, State Key Laboratory of Photocatalysis on Energy and Environment, College of Chemistry, Fuzhou University Fuzhou 350116 China klliu@fzu.edu.cn xcwang@fzu.edu.cn; b Department of Applied Chemistry and Center for Emergent Functional Matter Science, National Yang Ming Chiao Tung University Hsinchu 300 Taiwan sungfuhung@nycu.edu.tw; c Guangdong Provincial Key Laboratory of Semiconductor Optoelectronic Materials and Intelligent Photonic Systems, School of Integrated Circuits, Harbin Institute of Technology Shenzhen 518051 China; d Department of Medicinal and Applied Chemistry, Kaohsiung Medical University Kaohsiung 807 Taiwan

## Abstract

Heterogeneous frustrated Lewis pairs (FLPs) have emerged as an effective strategy to transform catalytically inert supports into active sites for hydrogenation reactions. However, the practical application of FLP sites remains limited due to their random spatial distribution and limited capacity to activate H_2_. To address these challenges, we report the rational design of a highly effective FLP-based catalyst by anchoring isolated Rh atoms onto CeO_2_, achieving a hydrogenation rate of 35%/h for styrene, remarkably outperforming pristine CeO_2_ (2.74%/h). It is found that Rh species on CeO_2_ form interfacial Rh–O–Ce sites, which play a critical role in the heterolytic cleavage of H_2_ into Rh–H^*δ*−^ and O–H^*δ*+^ species. Upon light irradiation, the hydrogen spillover process is significantly promoted, enabling more efficient migration of activated hydrogen species to FLP sites and thereby facilitating H_2_ dissociation under mild conditions. Moreover, photoexcitation of CeO_2_ generates abundant transient FLPs on the surface, which serve as additional active sites for hydrogenation, leading to a substantial enhancement in photocatalytic activity. Similar synergistic effects are also observed when other semiconductor supports are employed, indicating the generality of this strategy. These findings provide a new strategy for designing synergistic dual-active-site systems that integrate interfacial metal-oxide sites with photoinduced FLPs for efficient photocatalytic hydrogenation reactions.

## Abstract 2

The hydrogen spillover process from Rh–O–Ce interface sites to on the CeO_2_ surface, and then participate in the hydrogenation reaction on photo-driven transient FLP sites, thereby significantly improving the performance of hydrogenation.

## Graphical abstract

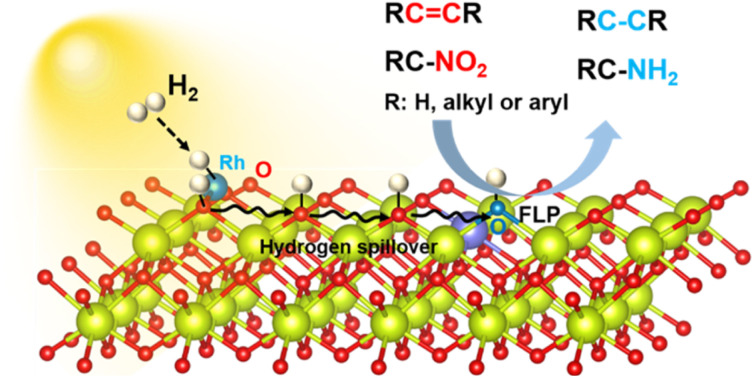

## Introduction

Heterogeneous frustrated Lewis pairs (FLPs) can effectively activate and transform small molecules through strong orbital interactions between FLP sites and reactant molecules, thereby converting catalytically inert oxides into active catalytic platforms.^1–3^ Despite substantial progress in heterogeneous FLP catalysis, their practical applications remain limited, mainly due to the random spatial distribution and inevitable agglomeration of FLP sites.^4–7^ Fortunately, the introduction of oxygen vacancies (V_Os_) into oxide supports not only enhances the acidity of Lewis acid sites but also strengthens the basicity of the corresponding Lewis base sites. This dual modulation significantly improves the catalytic performance of heterogeneous FLPs.^8–10^ However, although various strategies have been developed to generate oxygen vacancies on inert oxide surfaces, the precise construction and regulation of FLP sites *via* O_V_ engineering remain a formidable challenge.^11^

In photocatalysis, efficient charge separation can induce transient rearrangements of the electronic structure within photocatalysts.^12,13^ Photogenerated electron–hole pairs preferentially accumulate at different atomic sites—photogenerated holes locally decrease electron density, generating transient Lewis acid sites,^14–16^ whereas photogenerated electrons enrich electron density at neighboring sites, forming transient Lewis base or Lewis acid–base pairs.^17–19^ Accordingly, rational photocatalyst design offers a promising route for precise and dynamic construction of FLP sites under light irradiation.

FLP catalytic systems composed of sterically hindered electron donors and receptors represent one of the most successful classes of emerging hydrogenation catalysts.^20–22^ However, H_2_ dissociation over heterogeneous FLPs is often hindered by their electronic structure, and as a result, most reported FLP-mediated hydrogenation reactions require harsh reaction conditions. In this context, decorating highly reactive metals (*e.g.*, Rh and Pd) as atomically dispersed sites on oxide matrices has emerged as an effective strategy to enhance hydrogenation activity.^23–25^ The resulting metal–O–Ce interfacial structures can heterolytically activate H_2_ into metal–H^*δ*−^ and O–H^*δ*+^ species, which subsequently migrate onto the oxide surface where hydrogen addition occurs *via* a hydrogen spillover process.^7,26–28^ By physically decoupling H_2_ activation and hydrogenation sites, hydrogen spillover offers a unique opportunity to activate heterogeneous FLPs for efficient catalytic hydrogenation.^29–31^ On this basis, we reason that semiconductor-supported single atom catalysts can further benefit from the synergistic effects of hydrogen spillover and light irradiation, enabling enhanced catalytic hydrogenation under mild conditions.

In this work, we demonstrate that anchoring isolated Rh atoms onto CeO_2_ effectively overcomes the intrinsic limitations of heterogeneous FLPs, thereby significantly enhancing the photocatalytic hydrogenation performance of CeO_2_ under mild conditions. The resulting Rh_1_/CeO_2_ catalyst readily transforms catalytically inert FLP sites into highly active hydrogenation centers and exhibits a substantially higher photocatalytic activity for styrene hydrogenation compared with pristine CeO_2_. Mechanistic investigations reveal that isolated Rh species form interfacial Rh–O–Ce sites, which are responsible for the heterolytic activation of H_2_. Under light irradiation, two cooperative effects are observed: (i) photo-enhanced hydrogen spillover facilitates the migration of activated H species from Rh–O–Ce interfaces to FLP sites on CeO_2_, and (ii) photoexcitation of CeO_2_ generates a high density of transient surface FLPs. The synergistic interplay between these two photoinduced processes significantly improves both the activity and stability of photocatalytic hydrogenation. Notably, similar synergistic effects are also observed when other semiconductor supports are employed, indicating the generality of this strategy. This study establishes a synergistic dual-active-site concept—integrating interfacial metal-oxide sites with photoinduced FLPs—as an effective and broadly applicable approach for enhancing photocatalytic hydrogenation reactions.

## Results and discussion

**Fig. 1:**
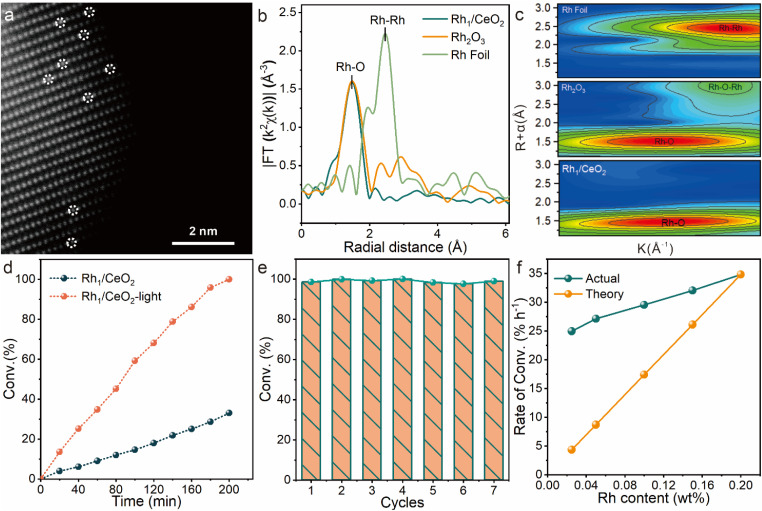
(a) HAADF-STEM image of the Rh_1_/CeO_2_ catalyst, (b) Fourier-transform EXAFS spectra of Rh_1_/CeO_2_ and references. (c) WT-EXAFS spectra of Rh_1_/CeO_2_ and references. (d) Catalytic performance of hydrogenation of styrene on Rh_1_/CeO_2_ under light irradiation. (e) Catalytic durability of Rh_1_/CeO_2_ in the hydrogenation of styrene. (f) The conversion of styrene within 1 h over Rh_1_/CeO_2_ with different loadings of Rh. Reaction conditions: 10 mL CH_3_CN; 2 µmol Rh; 2 mmol styrene; 303 K; 0.1 MPa H_2_; and a 300 W Xenon lamp (*λ* > 300 nm) as the light source.

The model CeO_2_ photocatalyst used in this study was synthesized *via* the hydrothermal method (Fig. S1).^32^ Isolated Rh species was subsequently deposited onto the as-prepared CeO_2_ using NaOH as the precipitating agent, yielding the Rh_1_/CeO_2_ catalyst.^33^ As shown in Fig. 1a and S2, high-angle annular dark-field scanning transmission electron microscopy (HAADF-STEM) images clearly confirm the atomic dispersion of Rh species on the fresh Rh_1_/CeO_2_ catalyst, with no evidence of Rh nanoparticle formation (Fig. S3). The atomic dispersion of Rh was further corroborated by *in situ* FTIR spectroscopy following CO pre-adsorption (Fig. S4). Only characteristic symmetric and asymmetric stretching vibrations of Rh(CO)_2_^+1^*gem*-dicarbonyl species were observed at around 2070 and 2000 cm^−1^, respectively, indicating the presence of isolated Rh sites.^34^ The local coordination environment of Rh atoms was further elucidated by X-ray absorption spectroscopy (XAS) measurements (Fig. 1b, c, S5–S7, S10 and Table S1). Wavelet transform (WT) contour plots of the Rh K-edge exhibit a single dominant feature at 5.5 Å^−1^, which can be assigned to Rh–O scattering. Notably, no Rh–Rh contributions were detected in either the WT or Fourier-transformed (FT) extended X-ray absorption fine-structure (EXAFS) spectra. Quantitative EXAFS fitting reveals a Rh–O coordination number close to four, further confirming the absence of Rh–Rh interactions (Fig. S7 and Table S1).

CeO_2_-based catalysts are well documented to be effective for alkene hydrogenation. Accordingly, styrene hydrogenation was selected as a model reaction to evaluate both the photocatalytic and thermocatalytic performance of Rh_1_/CeO_2_. As illustrated in Fig. 1d, S8 and S9, incorporation of isolated Rh species leads to a clear enhancement in catalytic activity compared with pristine CeO_2_; however, under dark conditions, the activity remains relatively limited. Remarkably, upon light irradiation, the Rh_1_/CeO_2_ catalyst exhibits a pronounced enhancement in both activity and stability, achieving 100% styrene conversion within 3 hours at a Rh:styrene molar ratio of 1 : 1000. This performance is approximately three times higher than that observed under dark conditions. Moreover, the catalyst maintains high activity over seven consecutive reaction cycles without noticeable deactivation (Fig. 1e), demonstrating excellent durability during photocatalytic hydrogenation. CO adsorption measurements conducted after cycling confirm that Rh species remain atomically dispersed, indicating the structural robustness of the Rh_1_/CeO_2_ catalyst (Fig. S11). Collectively, these results demonstrate that light irradiation plays a critical role in enhancing the hydrogenation performance of semiconductor-based catalysts.

Given that the Rh_1_/CeO_2_ catalyst contains two potential hydrogenation sites—Rh–O–Ce interfacial sites and FLP sites on CeO_2_—we next evaluate the individual catalytic contributions of these sites prior to mechanistic analysis of the photo-enhanced effect. As shown in Fig. 1f, S12 and S13, the Rh loading was systematically varied to investigate the role of Rh–O–Ce interfacial sites. Although decreasing the Rh loading leads to a reduction in overall catalytic activity, the decrease is notably non-linear. Specifically, when the Rh loading is reduced from 0.2 wt% to 0.025 wt% (Table S2), the catalytic activity does not decrease proportionally; instead, 71.65% of the activity observed at 0.20 wt% Rh is retained. This behavior indicates a substantial contribution from the CeO_2_ support to the hydrogenation process. Considering that pristine CeO_2_ is unable to efficiently activate H_2_ under mild conditions, the high hydrogenation activity of Rh_1_/CeO_2_ is predominantly attributed to hydrogen spillover, wherein H species activated at Rh–O–Ce interfacial sites migrate onto the CeO_2_ surface and participate in subsequent hydrogenation reactions.

**Fig. 2:**
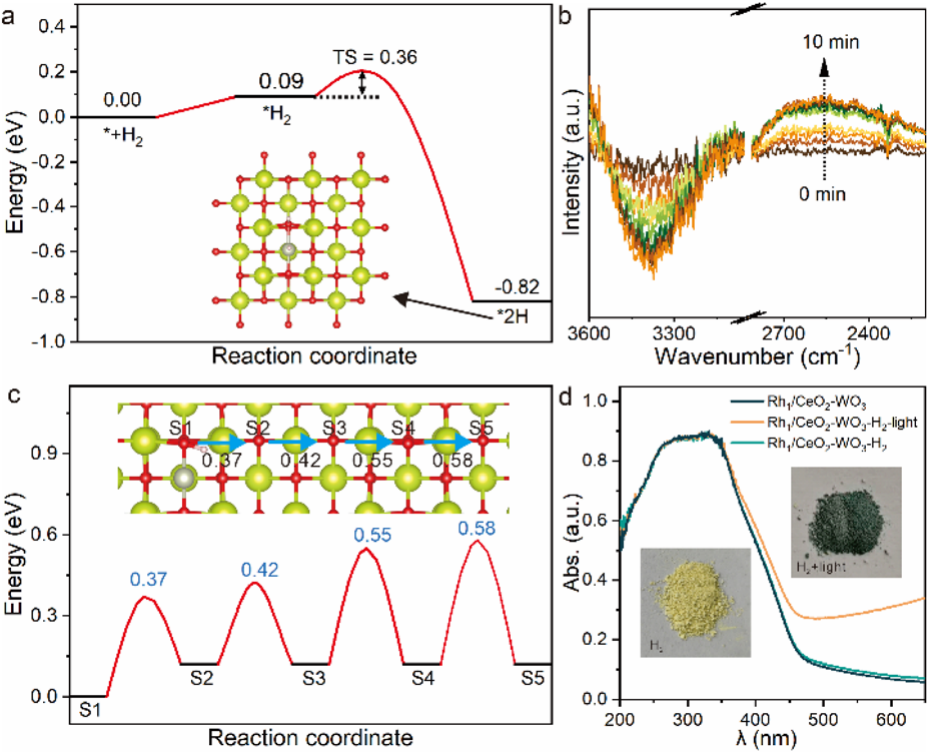
(a) Energy barriers of transition states and optimized structures for H_2_ activation on the Rh_1_/CeO_2_(100) surface. (b) *In situ* D_2_-FTIR spectra of Rh_1_/CeO_2_. (c) Energy barriers of transition states and optimized structures for the transfer of activated H on the Rh_1_/CeO_2_(100) surface. (d) UV-vis DRS spectra and photographs of the physical mixture of WO_3_ and CeO_2_ before and after treated with H_2_ and UV.

To further elucidate the mechanism of H_2_ activation on Rh_1_/CeO_2_, density functional theory (DFT) calculations were performed. As shown in Fig. 2a and S14, H_2_ adsorbed on isolated Rh sites is readily activated *via* a heterolytic cleavage pathway. In this process, one hydrogen atom migrates to a neighboring oxygen atom to form O–H^*δ*+^ species, while the other hydrogen atom remains bound to the Rh center as H^*δ*−^. This heterolytic H_2_ activation step is exothermic by 0.91 eV and proceeds with an activation barrier of 0.36 eV, indicating that hydrogen activation at the Rh–O–Ce interfacial sites is thermodynamically and kinetically favorable. *In situ* Fourier transform infrared (FT-IR) spectroscopy was subsequently employed to experimentally validate the theoretical predictions (Fig. 2b). Upon introduction of D_2_, a distinct absorption peak emerged at around 2500 cm^−1^, corresponding to the stretching vibration of –OD species. With prolonged D_2_ exposure, the characteristic –OH stretching peak in the range of 3200–3600 cm^−1^ gradually diminished, while the –OD signal became increasingly pronounced.^35^ In contrast, no –OD-related signals were detected for pristine CeO_2_ under identical D_2_ treatment conditions (Fig. S15). These observations unequivocally demonstrate that the Rh–O–Ce interfacial structure enables heterolytic H_2_ activation into H^*δ*−^ and H^*δ*+^ species under mild conditions, whereas CeO_2_ alone lacks this capability.

To gain further insight into the migration of activated hydrogen species from Rh–O–Ce interfacial sites to FLP sites on CeO_2_, DFT calculations were conducted to evaluate the hydrogen spillover process. As shown in Fig. 2c and S16–S19, the activated hydrogen atoms can readily migrate across the CeO_2_ surface by overcoming small energy barriers ranging from 0.37 to 0.58 eV, which are readily accessible under reaction conditions. Experimental evidence for hydrogen spillover was obtained using physical mixtures of Rh_1_/CeO_2_ and WO_3_ subjected to H_2_ treatment under light irradiation.^23^ As shown in Fig. 2d, the Rh_1_/CeO_2_-WO_3_ mixture exhibits a rapid color change from yellow to dark blue, indicative of hydrogen-induced reduction of WO_3_ and confirming the migration of activated hydrogen species from Rh_1_/CeO_2_ to WO_3_. In contrast, this spillover-induced color change is significantly suppressed in the absence of light irradiation or when atomically dispersed Rh atoms are not present (Fig. 2d and S20). Furthermore, H_2_ temperature-programmed reduction (H_2_-TPR) analysis reveals that the reduction of CeO_2_ is markedly accelerated by hydrogen spillover originating from Rh–O–Ce interfacial sites (Fig. S21). Taken together, these theoretical and experimental results confirm that hydrogen spillover from Rh–O–Ce interfacial sites to FLP sites on CeO_2_ is both energetically feasible and kinetically accessible, and that light irradiation plays a critical role in facilitating this process.

**Fig. 3:**
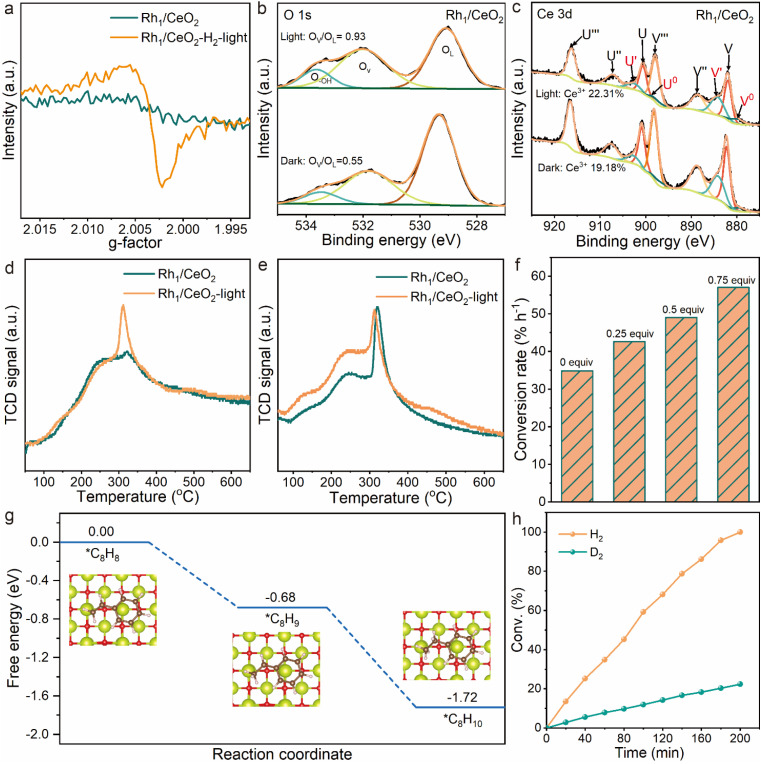
(a) *In situ* EPR spectra of Rh_1_/CeO_2_ before and after light irradiation. The XPS binding energy of (b) O 1s and (c) Ce 3d. (d) CO_2_-TPD and (e) NH_3_-TPD profiles of Rh_1_/CeO_2_ adsorbed with CO_2_/NH_3_ gas before and after treatment with light irradiation (Rh_1_/CeO_2_-light). (f) The reactivity of styrene hydrogenation over the physical mixture of Rh_1_/CeO_2_ and pure CeO_2_. (g) Stepwise hydrogenation (from 1H to 2H) of styrene. (h) The H_2_/D_2_ isotopic study of styrene hydrogenation catalyzed by Rh_1_/CeO_2_. Reaction conditions: 10 mL CH_3_CN; 2 µmol Rh; 2 mmol styrene; 303 K; 0.1 MPa H_2_ or D_2_; and a 300 W Xenon lamp (*λ* > 300 nm) as the light source.

Considering that light irradiation can induce the formation of transient FLPs on semiconductor surfaces, we hypothesized that the excellent hydrogenation activity originates from photo-generated transient FLPs serving as the active sites for styrene hydrogenation. To validate this hypothesis, *in situ* electron paramagnetic resonance (EPR) was employed to monitor changes in the electronic structure under light irradiation. As shown in Fig. 3a and S22, upon light irradiation at room temperature, Rh_1_/CeO_2_ exhibits a distinct EPR signal at a g-value of 2.003, which is characteristic of oxygen vacancies formed through the photo-reduction of CeO_2_. In contrast, no discernible EPR signal is observed in the absence of light irradiation. This comparison confirms that light irradiation induces significant electronic restructuring in CeO_2_, consistent with previous reports.^36^ More importantly, *in situ* X-ray photoelectron spectroscopy (XPS) reveals that light irradiation leads to the transformation of 22.09% of lattice oxygen (O_L_) into oxygen-vacancy-associated oxygen species, which act as Lewis acid sites (Fig. 3b and S23a). Correspondingly, the Ce(iv) species adjacent to these oxygen vacancies are partially reduced to Ce(iii), generating transient Lewis base sites (Fig. 3c and S23b). These results collectively provide direct evidence for the photoinduced formation of transient Lewis acid–base pairs on the CeO_2_ surface.

To directly probe the formation of transient FLPs under light irradiation, CO_2_ and NH_3_ are used as molecular probes for Lewis base and Lewis acid sites, respectively. As shown in Fig. 3d, CO_2_ temperature programmed desorption (CO_2_-TPD) profiles of Rh_1_/CeO_2_ exhibit two desorption peaks in the temperature ranges of 200–300 °C and 300–450 °C, attributable to CO_2_ adsorption on Lewis base sites associated with oxygen vacancies. Upon light irradiation, while the desorption temperatures remain unchanged, the total amount of desorbed CO_2_ increases significantly, indicating that light irradiation increases the number of Lewis base sites without altering their intrinsic absorption strength. Similarly, NH_3_ adsorption–desorption measurements exhibit analogous behavior (Fig. 3e), further confirming that light irradiation enhances the population of Lewis acid sites while preserving their adsorption characteristics.

To further verify that photogenerated transient FLPs serve as the active sites for hydrogenation by spilled hydrogen species, we investigated the relationship between catalytic activity and the density of transient FLPs by introducing additional CeO_2_ into the reaction system. Specifically, pristine CeO_2_ is physically mixed with Rh_1_/CeO_2_. As shown in Fig. 3f and S24, the hydrogenation activity increases by ∼63.82% upon the addition of 0.75 equiv. of CeO_2_. Moreover, the catalytic activity increases progressively with increasing amounts of added CeO_2_. These results clearly demonstrate that the spilled hydrogen species participate in hydrogenation reactions at photo-induced transient FLP sites on CeO_2_.

To gain deeper insight into the role of spilled hydrogen species in FLP-mediated hydrogenation, DFT calculations were performed to evaluate the Gibbs free energy changes (Δ*G*) associated with each elementary hydrogenation step. As shown in Fig. 3g and S25–S27, the calculated Δ*G* values for hydrogen addition on FLP sites are strongly negative, indicating that styrene hydrogenation on these sites is thermodynamically favorable. Then, kinetic isotope effect (KIE) measurements and *in situ* FT-IR analysis were performed to further confirm the involvement of spilled hydrogen species. When D_2_ was used as the hydrogen source, a pronounced KIE value of 4.43 was observed (Fig. 3h), indicative of a primary isotope effect and confirming that proton transfer involving spilled hydrogen species plays a key role in the rate-determining hydrogenation steps. In addition, the signal of –OD on Rh_1_/CeO_2_ gradually disappeared after the introduction of styrene gas, indicating that *D*^*δ*+^ was involved directly in the hydrogenation of styrene (Fig. S28). Together, these theoretical and experimental results unambiguously establish that FLP sites on the CeO_2_ surface serve as the active centers for styrene hydrogenation, in full agreement with the mechanistic insights derived from hydrogen spillover and photo-induced FLP formation.

**Fig. 4:**
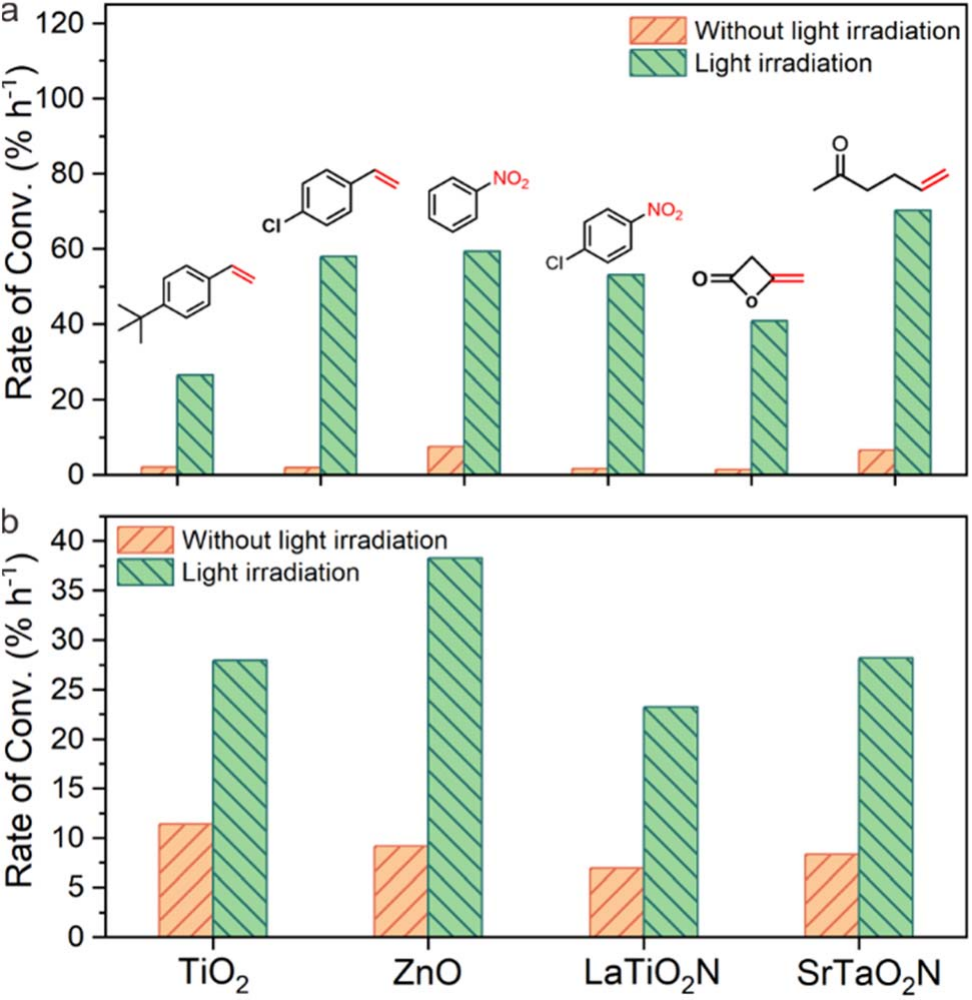
(a) Conversion rates for the photocatalytic hydrogenation of other unsaturated compounds over Rh_1_/CeO_2_. (b) Conversion rates of TiO_2_, ZnO, LaTiO_2_N and SrTaO_2_N. Reaction conditions: 10 mL CH_3_CN; 2 µmol Rh; 2 mmol styrene; 303 K; 0.1 MPa H_2_; and a 300 W Xenon lamp (*λ* > 300 nm) as the light source.

The above studies collectively demonstrate that photo-induced transient FLPs generated under light irradiation are essential for achieving the enhanced hydrogenation activity. We thus expect that the photo-enhanced catalytic hydrogenation should promote the hydrogenation of a wide range of unsaturated compounds. Experimentally, Rh_1_/CeO_2_ also displays excellent catalytic performance in the hydrogenation of other unsaturated compounds, such as 4-*tert*-butylstyrene, 4-chlorostyrene, nitrobenzene, 4-chloronitrobenzene, diketene, and allylacetone, with activities of 26.4%//h, 58.0%/h, 59.4%/h, 53.1%/h, 40.9%/h, and 70.2%/h (Fig. 4a and S30), respectively. Based on this understanding, we further sought to extend this concept to other semiconductor-supported, isolated Rh catalysts to establish the universal principles of photo-driven transient FLPs for catalytic hydrogenation. Following a similar synthetic strategy used for Rh_1_/CeO_2_, isolated metal atoms were deposited onto a series of semiconductor supports, including TiO_2_, ZnO, LaTiO_2_N, and SrTaO_2_N, *via* reductive deposition and subsequent deposition–precipitation methods (Fig. S30–S33). The hydrogenation performance of the resulting catalysts was evaluated using styrene as a probe substrate. As shown in Fig. 4b and S34, all semiconductor-supported catalysts exhibit markedly enhanced hydrogenation activity toward the alkene products compared with the corresponding metal-on-carbon reference catalyst under identical conditions. These results demonstrate that the synergistic interplay between isolated metal sites—responsible for efficient H_2_ activation—and photo-driven transient FLPs—serving as hydrogenation centers—is a general and effective strategy for turning catalytically inert semiconductor supports into highly active hydrogenation catalysts under mild reaction conditions.

## Conclusions

In summary, this work demonstrates a synergistic strategy to overcome the intrinsic limitations of heterogeneous frustrated Lewis pairs (FLPs) for catalytic hydrogenation under mild conditions. By anchoring isolated Rh species onto CeO_2_, well-defined Rh–O–Ce interfacial sites are constructed that enable efficient heterolytic H_2_ activation into Rh–H^*δ*−^ and O–H^*δ*+^ species. Under light irradiation, the hydrogen spillover process from these interfacial sites to FLP sites on the oxide surface is markedly promoted, while photoexcitation of CeO_2_ simultaneously generates abundant transient FLPs. The cooperative interplay between photo-enhanced hydrogen spillover and photo-induced FLP formation results in substantial enhancement in photocatalytic hydrogenation activity, exemplified by the markedly improved hydrogenation rate of a wide range of unsaturated compounds compared with pristine CeO_2_. Importantly, the observation of similar synergistic effects on other semiconductor supports highlights the general applicability of this approach. This study establishes a versatile dual-active-site design principle that integrates interfacial metal-oxide sites with photoinduced FLPs, providing a new and broadly applicable pathway for the rational design of efficient photocatalytic hydrogenation systems.

## Author contributions

Jin Lin: writing – original draft, data curation, investigation; Shuanghui Chen: data curation, investigation; Kang-Shun Peng: investigation; Yung-Hsi Hsu: investigation; Shuchun Li: investigation; Longji Cui: investigation; Hansong Zhang: investigation; Yongjie Wang: investigation; Xue Feng Lu: investigation; Sibo Wang: investigation; Kunlong Liu: writing – review and editing, project administration, supervision, funding acquisition; Sung-Fu Hung: resources, writing – review and editing, supervision; Xinchen Wang: writing – review and editing, project administration, supervision, funding acquisition.

## Conflicts of interest

There are no conflicts to declare.

## Supplementary Material

SC-OLF-D6SC02736A-s001

## Data Availability

The data supporting this article have been included as part of the supplementary information (SI). Supplementary information is available. See DOI: https://doi.org/10.1039/d6sc02736a.
