# Supplementary material for: Photo-driven transient frustrated Lewis pairs for catalytic hydrogenation
Source: Chem Sci. 2026 Jun 25;17(32):15451–7. doi: 10.1039/d6sc02736a (PMC13321809; doi:10.1039/d6sc02736a)
Supplement: Supplementary file 1 [file SC-017-D6SC02736A-s001.pdf]

Supplementary Information (SI) for Chemical Science.
This journal is © The Royal Society of Chemistry 2026

Supplementary Information for

Photo-driven transient frustrated Lewis pairs for catalytic hydrogenation

Jin Lin,[1] Shuanghui Chen,[1] Kang-Shun Peng,[2] Yung-Hsi Hsu,[2] Shuchun Li,[1] Longji Cui,[1] Hansong Zhang,[3] Yongjie Wang,[3] Xue Feng Lu,[1] Sibo Wang,[1] Kunlong Liu,[1,] Sung-Fu Hung,[2,4,*] and Xinchen Wang[1,*]*

[1]State Key Laboratory of Chemistry for NBC Hazards Protection, State Key Laboratory of Photocatalysis on Energy and Environment, College of Chemistry, Fuzhou University, Fuzhou 350116, China

[2]Department of Applied Chemistry and Center for Emergent Functional Matter Science, National Yang Ming Chiao Tung University, Hsinchu 300, Taiwan

[3]Guangdong Provincial Key Laboratory of Semiconductor Optoelectronic Materials and Intelligent Photonic Systems, School of Integrated Circuits, Harbin Institute of Technology, Shenzhen 518051, China

[4]Department of Medicinal and Applied Chemistry, Kaohsiung Medical University, Kaohsiung 807, Taiwan

**Corresponding authors. Email: klliu@fzu.edu.cn, sungfuhung@nycu.edu.tw, xcwang@fzu.edu.cn*

Experimental Section

Materials. Rhodium(III) chloride trihydrate ($RhCl_3 \cdot 3H_2O$, 98%) were purchased from Innochem. Lithium hydroxide (anhydrous, NaOH, 98%) and Cerium nitrate hydrate ($Ce(NO_3)_3 \cdot 6H_2O$, 99.9%) were purchased from Shanghai Aladin Biochemical Technology Co. Ltd., China. Tungsten oxide (WO_3) and Ethanol (C_2H_5OH) were purchased from Sinopharm Chemical Reagent Co. Ltd., China. H_2 (99.99%), D_2 (99.999%), CO (99.999%), N_2 (99.999%) were purchased from Nan'an Chenggong Gas Co. Ltd., China. The water used in all experiment was deionized water (18.2 MΩ). All chemicals were used as received without further purification.

Synthesis of photocatalysts.

Synthesis of CeO_2. Cubic CeO_2 were prepared by the hydrothermal method. 1.74 g of $Ce(NO_3)_3 \cdot 6H_2O$ and 14.4g of NaOH were dissolved in 5 mL and 55mL of deionized water respectively. The $Ce(NO_3)_3$ solution was slowly dropped into the NaOH solution and stirred at room temperature for 30 minutes to obtain a purple slurry. The slurry was transferred to a 100 mL stainless steel autoclave with a polytetrafluoroethylene inner liner and heated at 453.15K for 24 hours. After the hydrothermal process, the sample was washed with deionized water and dried under vacuum at 333.15K for 12 hours to obtain Cubic CeO_2.

Synthesis of Rh_1/CeO_2. The Rh_1/CeO_2 catalyst was prepared by the deposition-precipitation (D-P) method. 600 mg of cubic CeO_2 were uniformly dispersed by ultrasound

in 250 ml of deionized water. The mixture was stirred at room temperature for 12 hours. Then, 600 μL of the pre-prepared 2 mg/mL $RhCl_3$ solution was slowly added to the system and stirred at room temperature for 12 hours. Subsequently, a concentrated NaOH solution was added to adjust the pH of the system to 14, and the mixture was stirred at room temperature for 12 hours. After the stirring was completed, the mixture was washed with deionized water until the pH was 7. The catalyst was then vacuum-dried at 333.15 k for 12 hours.

Photocatalytic reactions. *Catalytic hydrogenation reaction under dark conditions.* 2 μmol Rh was dispersed in 10 mL acetonitrile with stirring at 30 ^{o}C using a water bath equipped with circulating water (catalyst amount: 0.206 mg/L). The air in the system was removed by N_2 bubbling for 30 min. Then, 2 mmol of styrene was added to the suspension, and the hydrogenation reaction was carried out under a 0.1 MPa atmosphere of H_2.

Catalytic hydrogenation reaction under light conditions. The procedure was identical to that described above, except that the reaction solution was irradiated with a 300 W Xenon lamp light (λ> 300 nm) throughout the hydrogenation process (The vertical distance from the light source to the liquid surface is 16 cm). The wavelengths and power densities of the LED lights were tested by a NIST traceable light measurement systems (International Light Technologies). The potential heat effect was minimized by using a water bath equipped with circulating water. After the reaction, the products were analyzed by a gas chromatograph (Shimadzu GC-2030) equipped with an FID detector.

Characterization. TEM and STEM studies were performed on a TECNAI F30 transmission electron microscope at 300 kV. Power X-ray diffraction (XRD) experiments were performed on the Rigaku MiniFX600 with a Cu K_α X-ray radiation source (λ = 0.154056 nm) at a scan rate of 10 °/min. Electron paramagnetic resonance (EPR) spectra were recorded at 293.15 K using a Bruker A300. UV-Vis absorption spectra were collected using a UV-Vis-NIR spectrophotometer (Cary 5000, Agilent) with barium sulfate as the background correction standard. The UV absorption spectra were collected by use of a UV spectrophotometer (UV-2400PC, Shimadzu) with barium sulfate as a standard for the background correction. Inductively Coupled Plasma Optical Emission Spectrometer (ICP-OES) (Agilent 5110) is used to analyze the specific content of the Rh element in Rh_1/CeO_2.

XAS measurements. X-ray absorption spectroscopy (XAS) measurements were conducted using a custom-designed reactor at beamlines 17C of the Taiwan Light Source (TLS) and 44A and 32A of the Taiwan Photon Source (TPS), NSRRC. The XAS data were collected at the Rh K-edge (23220 eV). X-ray absorption near-edge structure (XANES) spectra were processed by subtracting the pre-edge background and normalizing the spectra using a spline fit via the ATHENA software package. The k2-weighted extended X-ray absorption fine structure (EXAFS) spectra were Fourier transformed over a k-range of 3-11 $Å^{-1}$. EXAFS fitting was subsequently carried out in the R range of 1~3 Å using the ARTEMIS program to verify the structural parameters.

Diffuse reflectance FTIR characterizations. *In situ* FTIR was carried out by using 100 mg catalyst that was pressed into a small disc and then transferred into an in-situ chamber of FTIR. The chamber was flushed with 1 bar Ar at room temperature ten times and then recorded by FTIR (Nicolet IS-50). After, CO (or D_2) was carried into the chamber for 30 min. In order to observe the adsorption state of the CO molecule, once again, the chamber was flushed with 1 bar Ar at room temperature ten times to removing the unadsorbed CO on the surface of the catalyst. The blank chamber with Ar was used for background correction.

***In-situ* EPR spectrometer analysis.** Electron paramagnetic resonance (EPR) spectra were recorded at 293 K using a Bruker A300. The sample was filled into a standard EPR quartz sample tube (outer diameter 4 mm) and were illuminated in situ with a UV light (100W, 365 nm). The experiment was conducted at room temperature with the cryostat temperature maintained at 293 K. The EPR experiment involved first collecting the spectrum without turning on the UV light, and then, after 15 minutes of UV light activation, spectra were collected again to compare the signal changes before and after photocatalysis, thus determining information about oxygen vacancies.

***In-situ* XPS spectrometer analysis.** The *In-situ* X-ray photoelectron spectroscopy (XPS) was carried out on Escalab Xi^+ high-performance electron spectrometer with Al Kα (hv = 1486.8 eV) as the excitation source. The continuous tunable wavelength light source was Self-made introduction-type 300W Xenon light fiber (320 nm-800 nm). In a typical

test, the dark environment binding energy was characterized with all lights turned off in the SAC chamber. Then the irradiation light source was turned on, the wavelength was set to 365 nm, and the test was conducted.

***In-situ* NH_3/CO_2-TPD spectrometer analysis.** *In-situ* NH_3/CO_2-TPD experiments were performed at REACT. In a typical experiment, a U-tube was loaded with 100 mg of sample atop a packed bed of quartz wool. The U-tube was affixed to the gas line in a heated reactor. A thermocouple probe was fixed inside the U-tube so that it came to contact with the top of the sample. The sample was first purged with He for 1 h at 313 K, then heated to 323 K under a 20 sccm flow of He. The sample was then treated with 20 sccm of 10% NH_3/CO_2 in He for 60 min under UV light to completely saturate with NH_3/CO_2. The sample was then purged with 20 sccm of He for 60 min to remove physisorbed NH_3/CO_2, then heated at 10 K/min under a flow of 20 sccm He to a maximum temperature of 1073 K.

Computational methods. All of density functional theory (DFT) calculations were conducted using the Vienna *ab initio* Simulation Package (VASP) with the projector augmented wave (PAW) method.[1-3] The exchange-correlation energy was described employing the Perdew-Burke-Ernzerhof (PBE) generalized gradient approximation (GGA) method.[4] The constructed cubic $CeO_2(100)$ surface consists of 1×2 unit cells, totaling four layers. We anchored a single Rh atom on the $CeO_2(100)$ surface and constructed oxygen vacancies (O_{Vs}), thereby achieving a Rh/$CeO_2(100)$ structure with oxygen vacancies. The bottom three layers of Rh/$CeO_2(100)$ were constrained to maintain the bulk crystal

structure. The vacuum space was set to 15 Å to avoid electronic interactions between adjacent cells. The cutoff energy was 500 eV. The convergence settings for energy and force during structure optimization were 1×10^{-5} eV/atom and 0.02 eV/Å, respectively. A Monkhorst mesh of 1 × 1 × 1 was utilized in k-point sampling.[5] A van der Waals (vdW) interaction was described by the DFT-D3 empirical correction.[6] The climbing image nudged elastic band (CINEB) method was employed to locate the minimum-energy paths, and force convergence standard is 0.03 eV $Å^{-1}$.[7] The transition states were verified using vibrational frequency calculations.

The Gibbs free energy change (ΔG) of styrene (C_8H_8) hydrogenation was computed using computational hydrogen electrode (CHE) model [8]:

$$\Delta G = \Delta E + \Delta E_{\mathrm{ZPE}} - T\Delta S$$

where ΔE is the electronic energy difference from DFT calculations. ΔE_{ZPE} and ΔS represent the change in zero-point energy and entropy respectively. T is the system temperature (298 K).

Supporting Figures

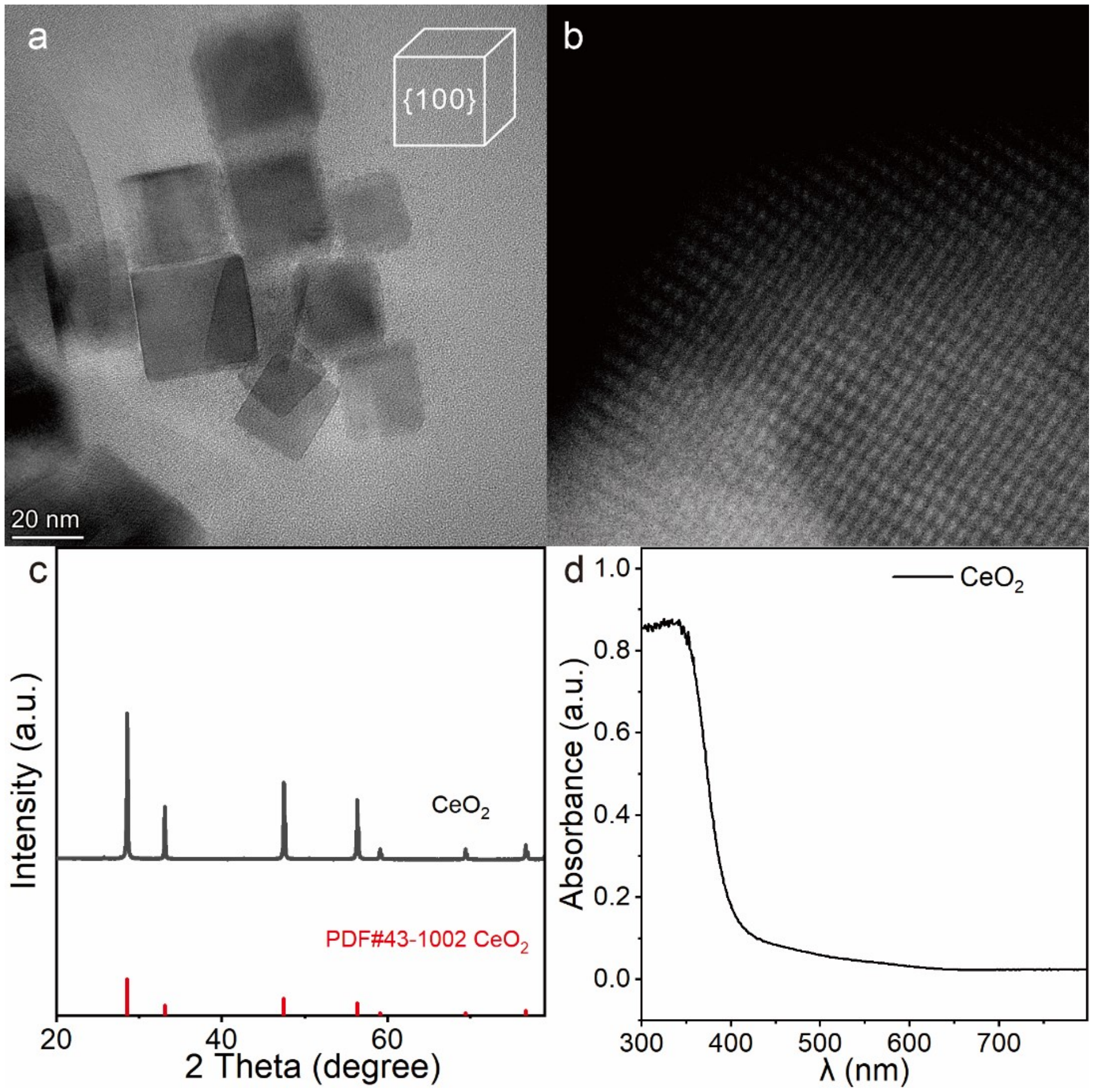

Figure S1. (a) TEM image, (b)Aberration-corrected high-angle annular dark-field scanning transmission electron microscopy (AC-HAADF-STEM) image, (c) XRD patterns and (d) UV-vis DRS of CeO_2.

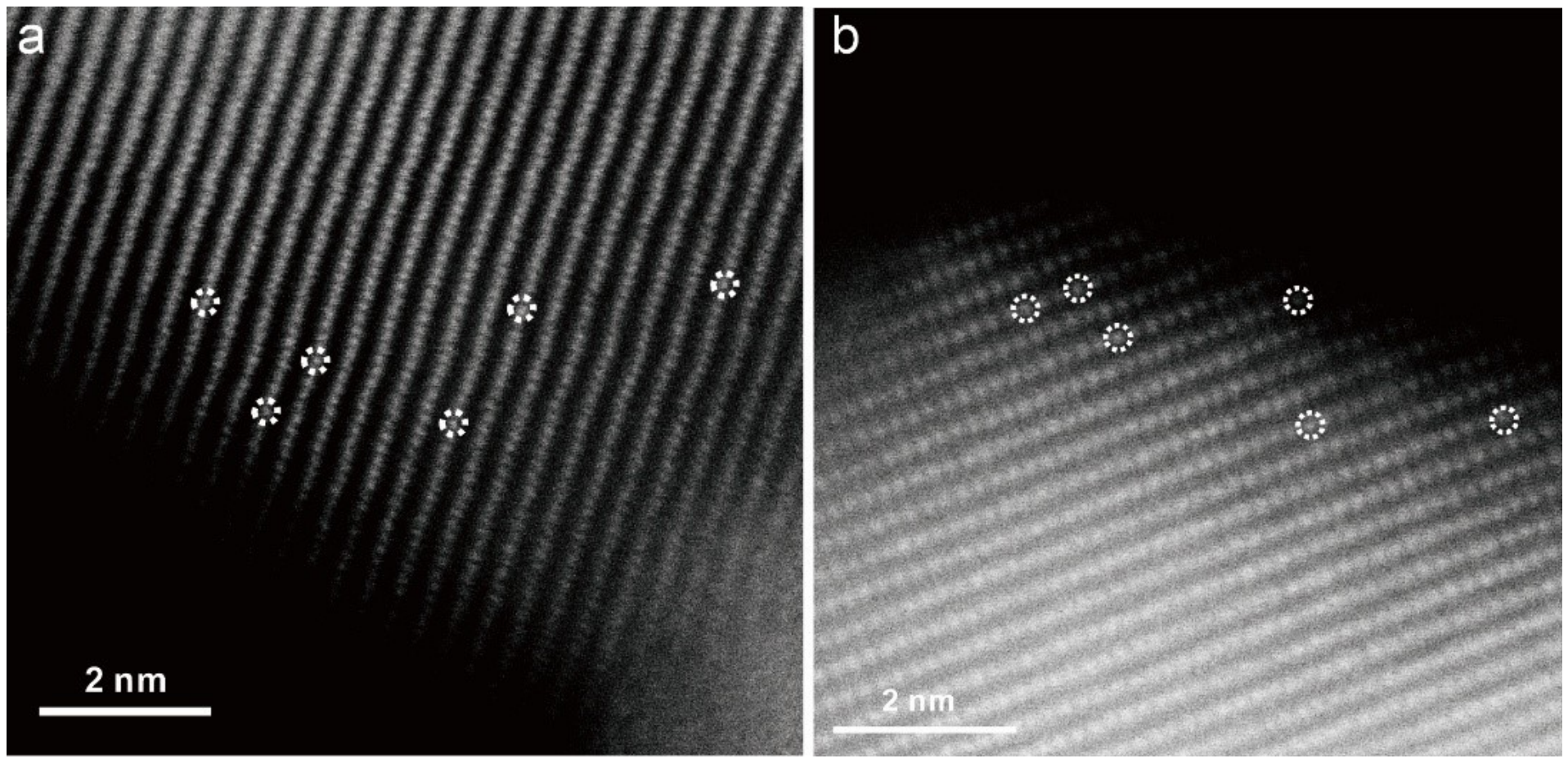

Figure S2. Aberration-corrected high-angle annular dark-field scanning transmission electron microscopy (AC-HAADF-STEM) image of Rh_1/CeO_2.

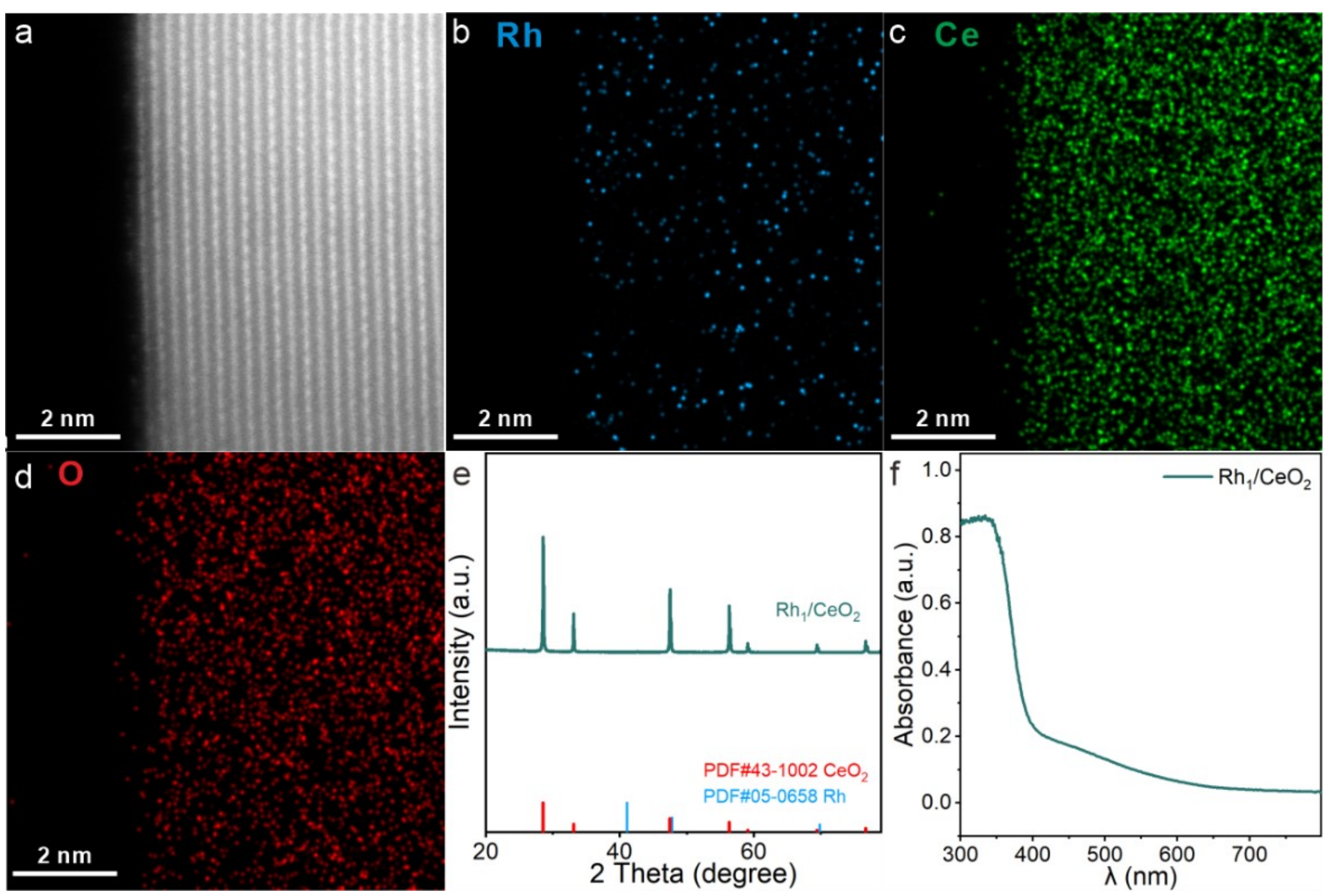

Figure S3. (a-d) AC-HAADF-STEM-EDS elemental mapping of Rh_1/CeO_2, (e) XRD patterns and (f) UV-vis DRS of Rh_1/CeO_2.

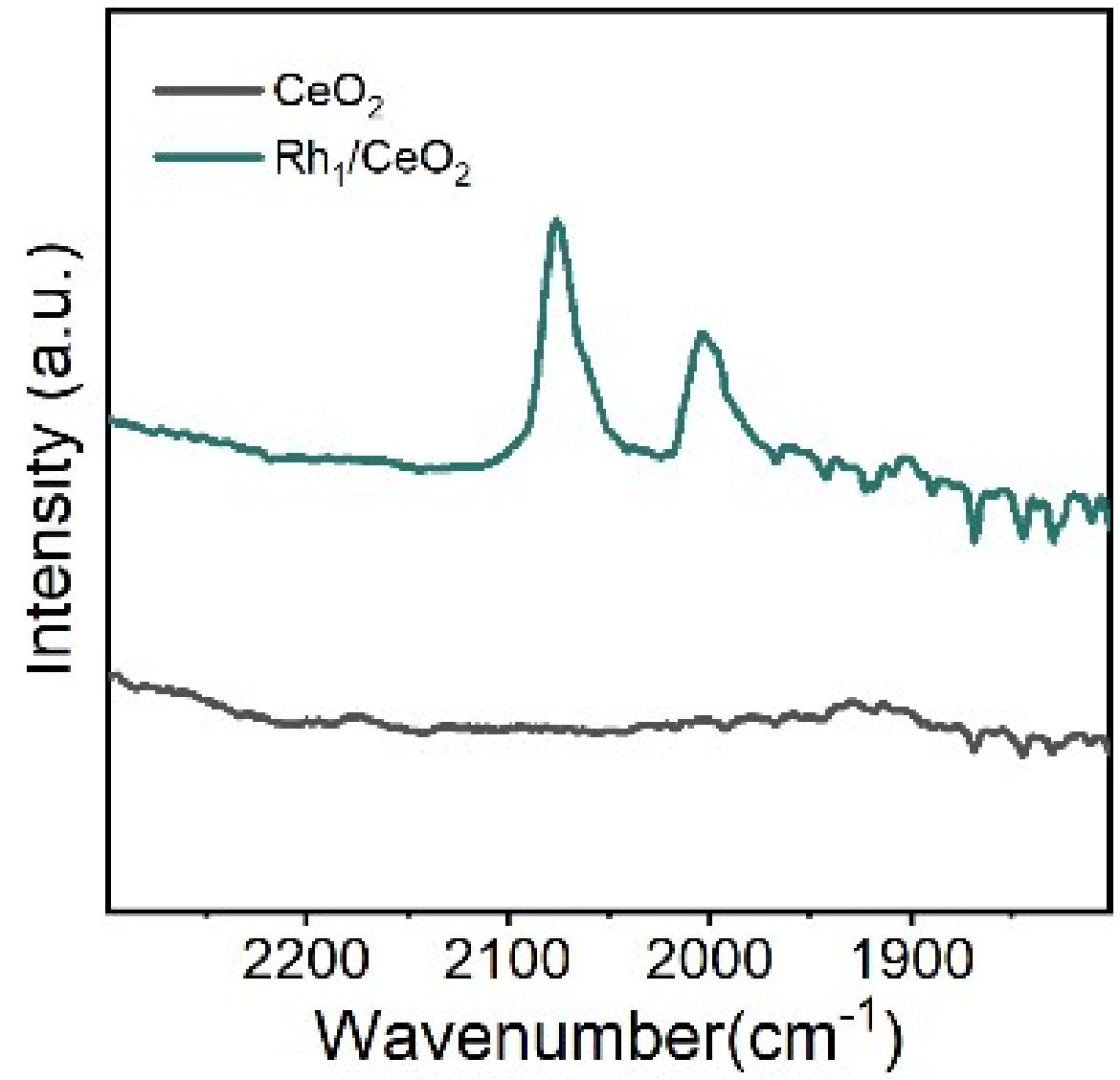

Figure S4. CO-FTIR curves for Rh_1/CeO_2 and CeO_2.

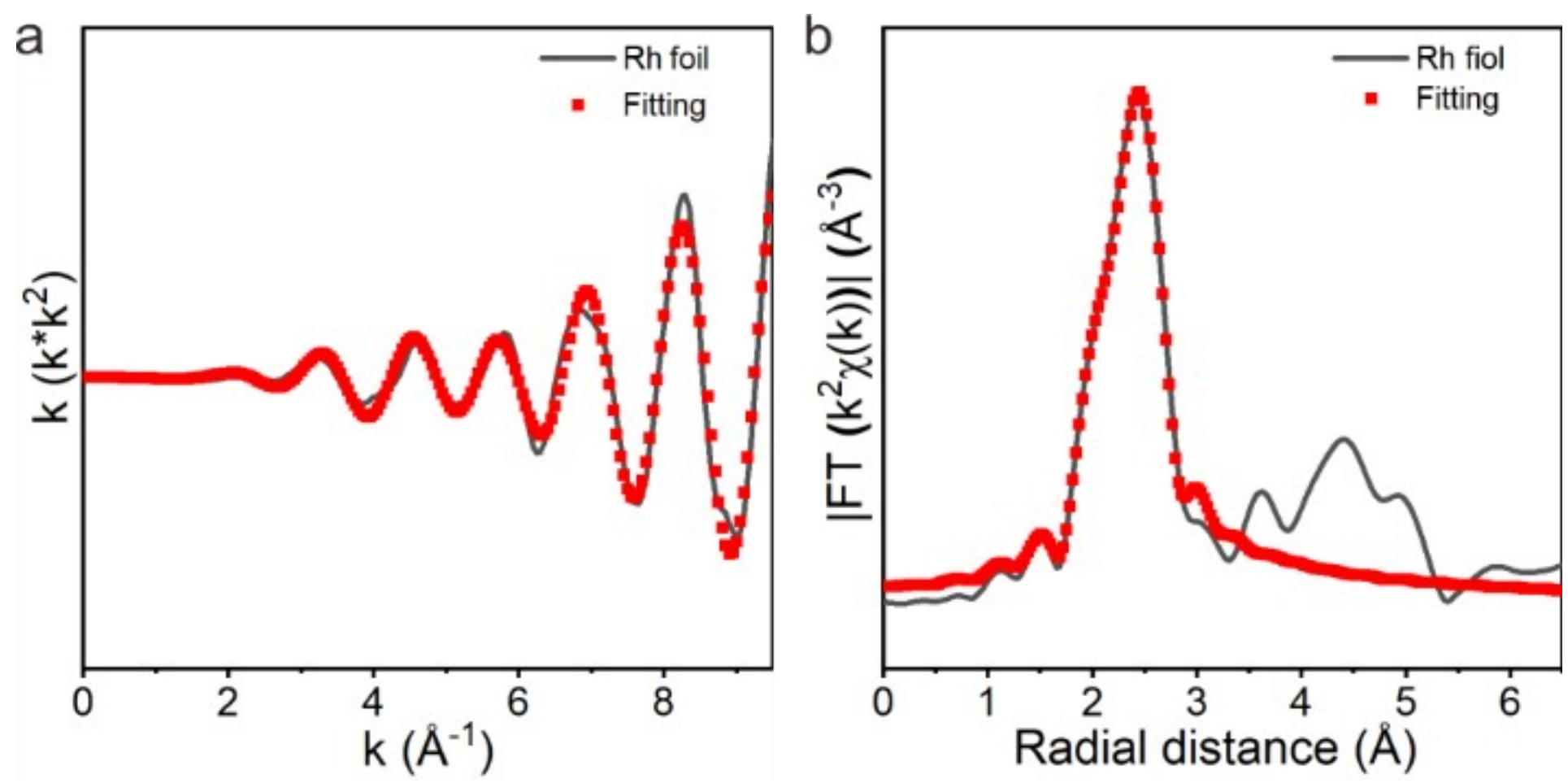

Figure S5. The fitted K-space and corresponding Fourier transformed R-space EXAFS spectra of Rh foil.

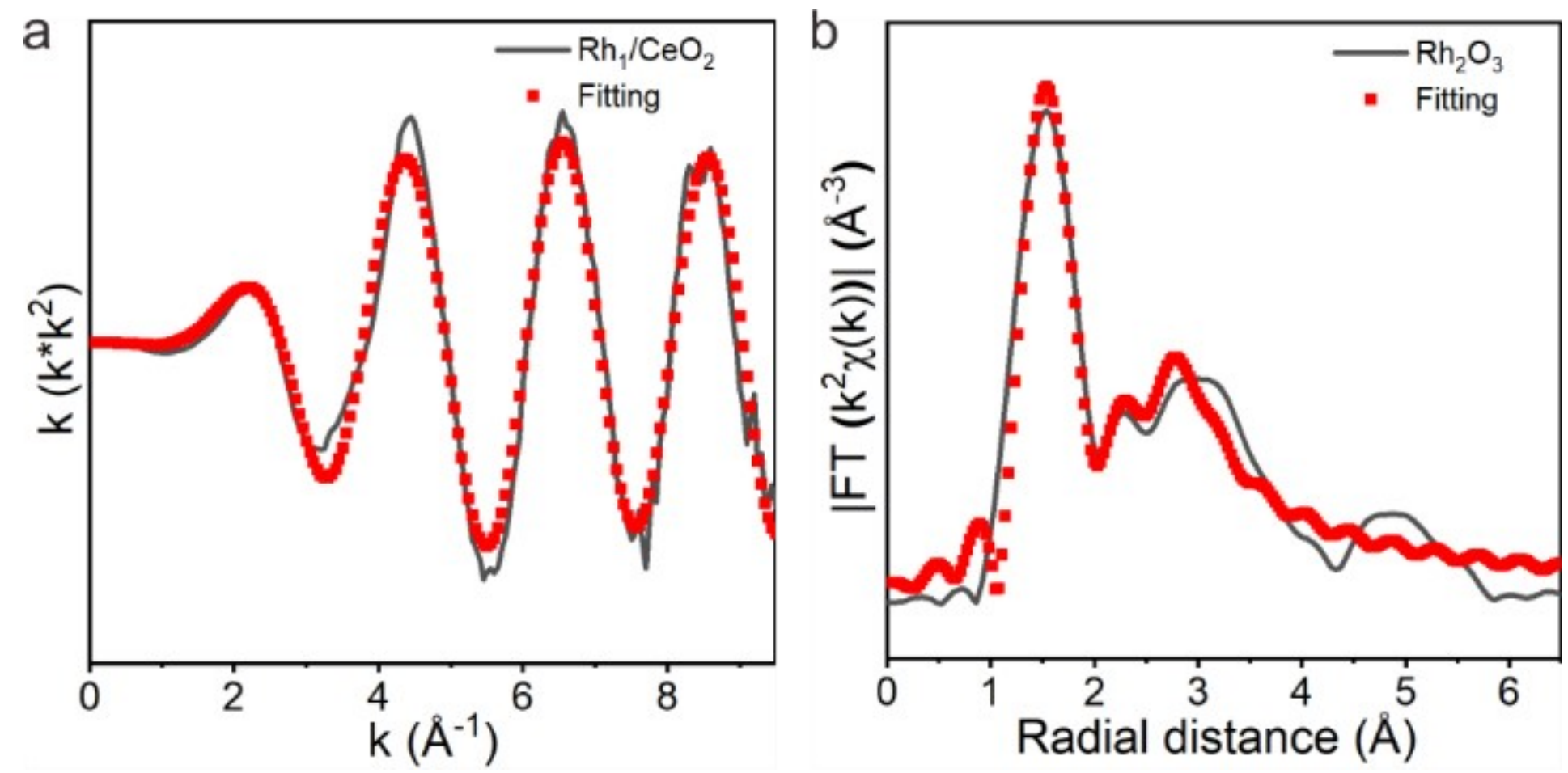

Figure S6. The fitted K-space and corresponding Fourier transformed R-space EXAFS spectra of Rh_2O_3.

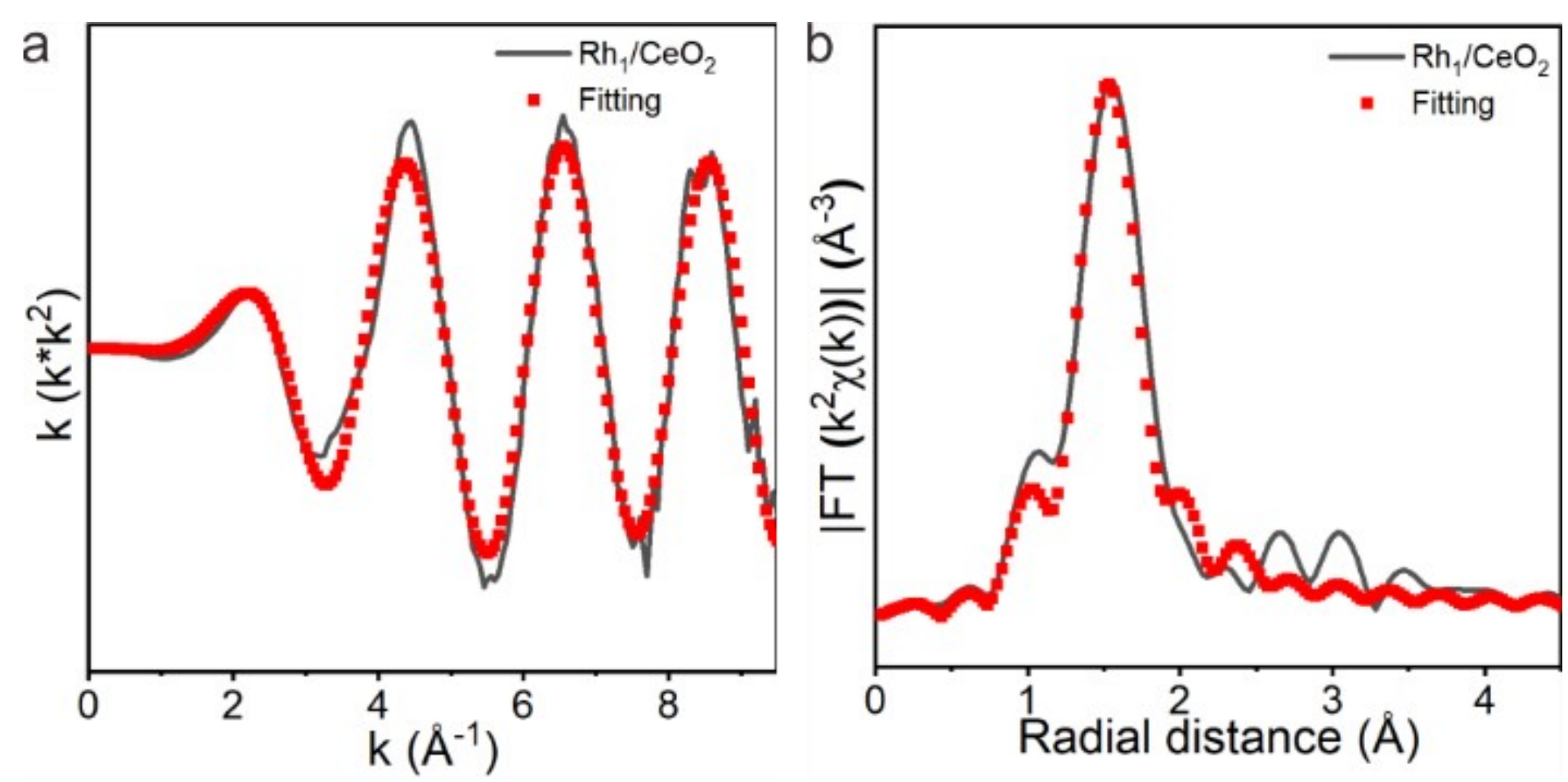

Figure S7. The fitted K-space and corresponding Fourier transformed R-space EXAFS spectra of Rh_1/CeO_2.

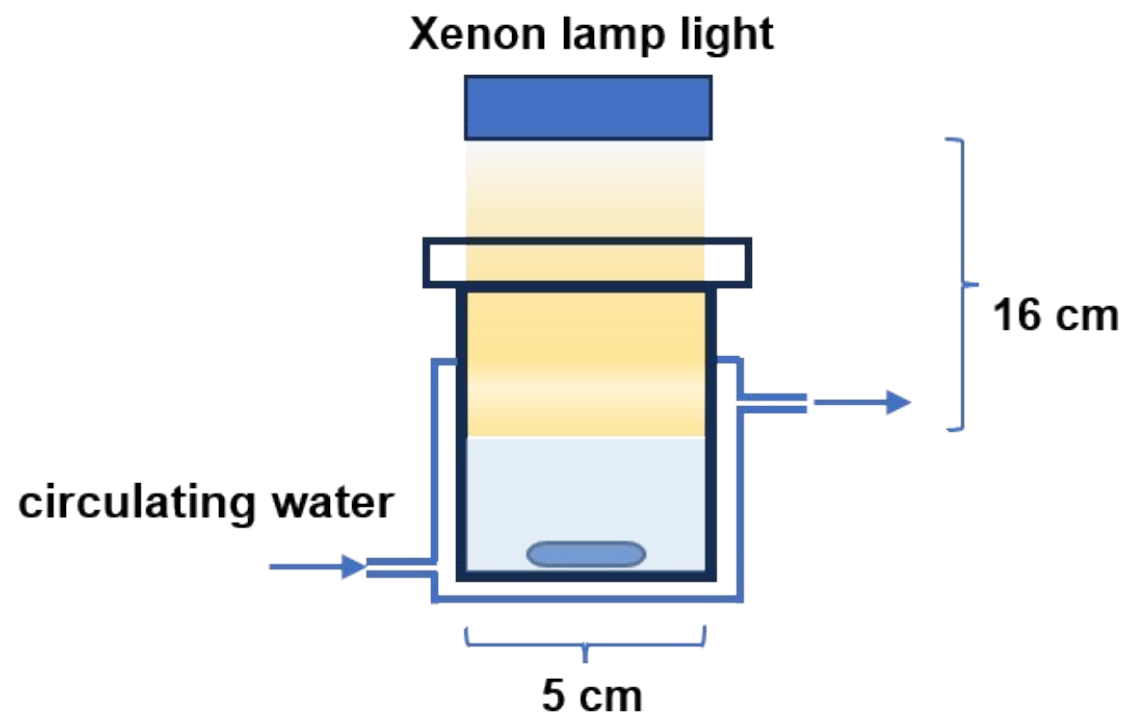

Figure S8. Schematic diagram of the photocatalytic reaction device

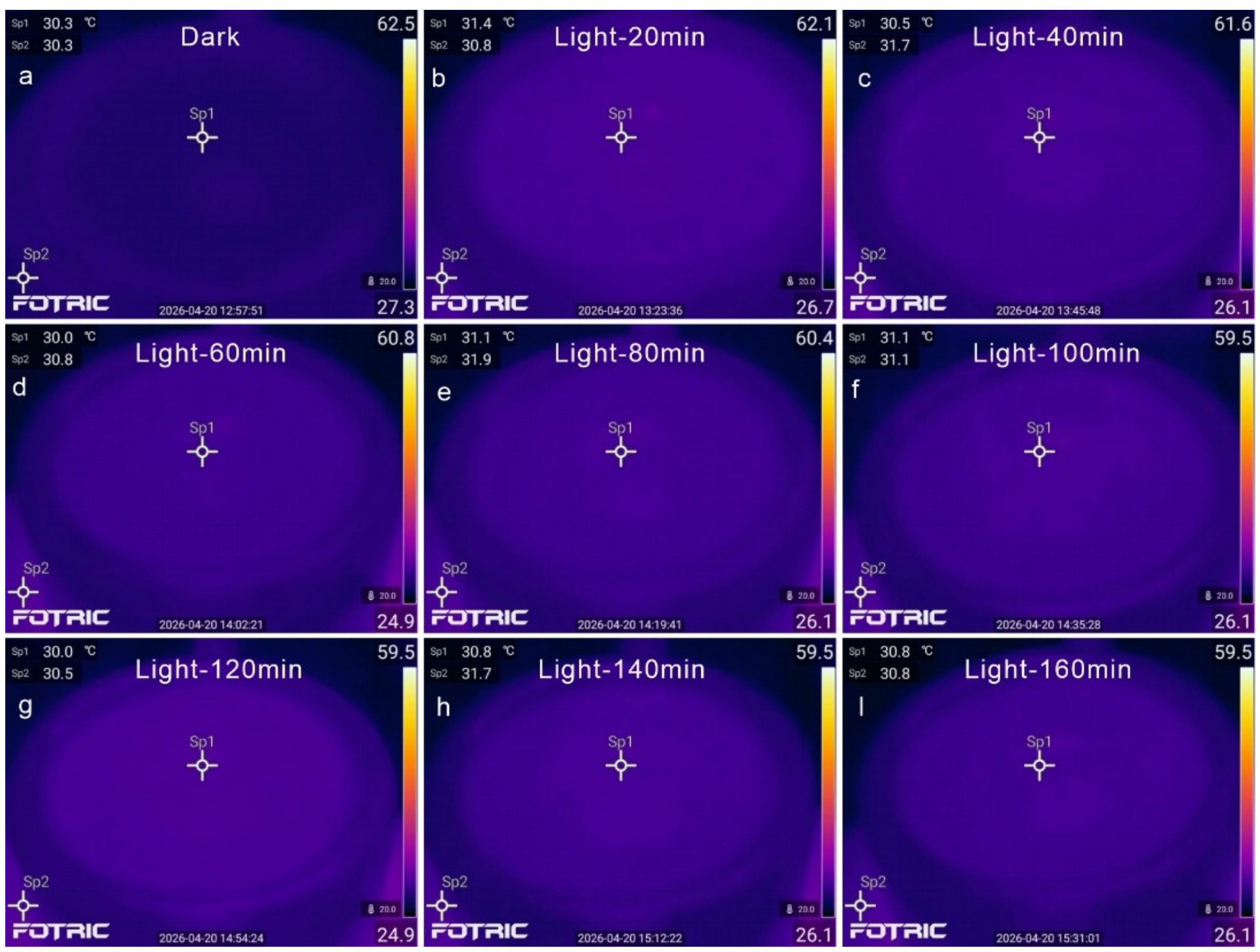

Figure S9. The actual surface temperature of the catalyst (a) before and (b-i) after irradiation.

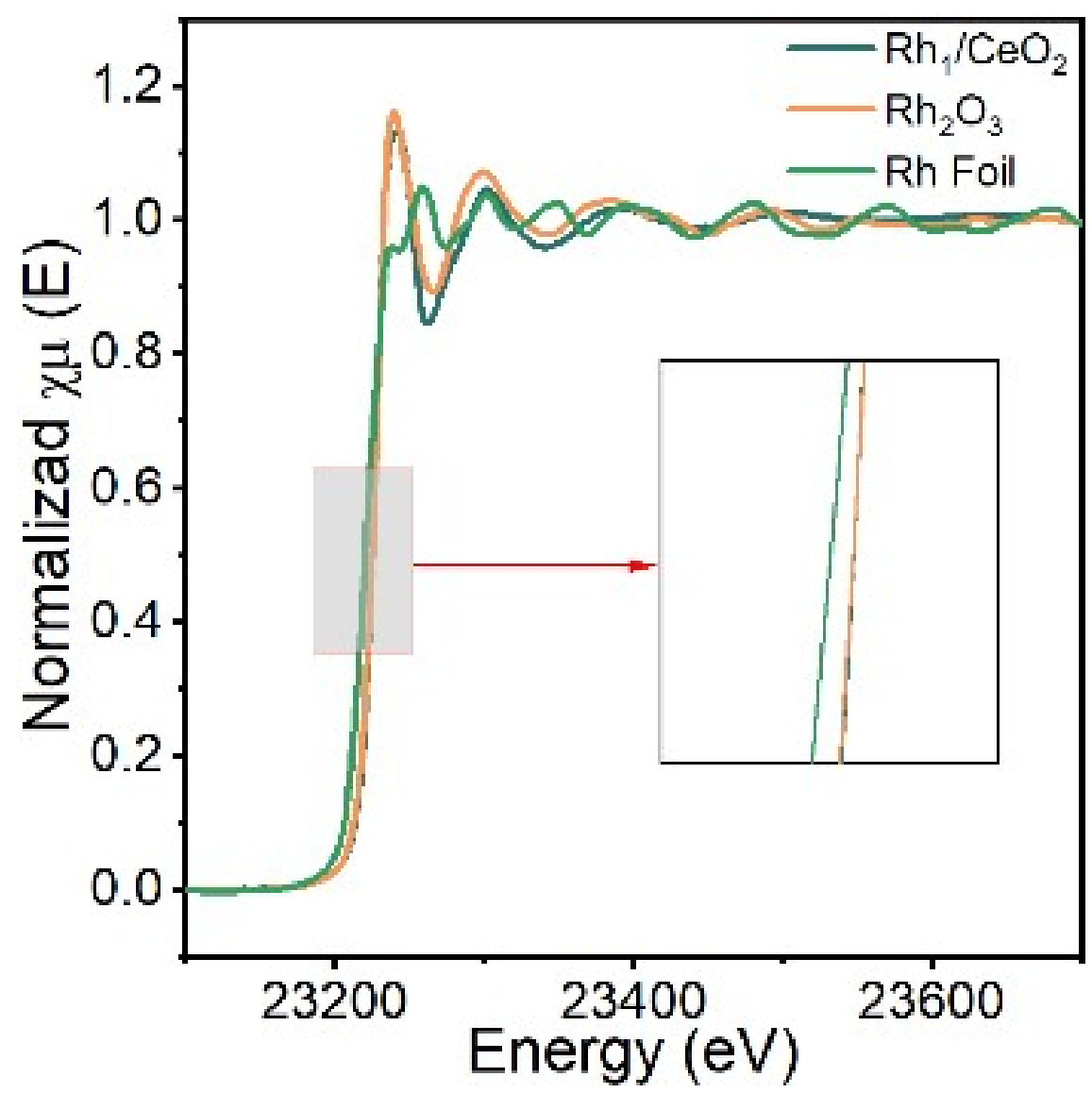

Figure S10. Normalized Rh K-edge XANES spectra of Rh_1/CeO_2 and references.

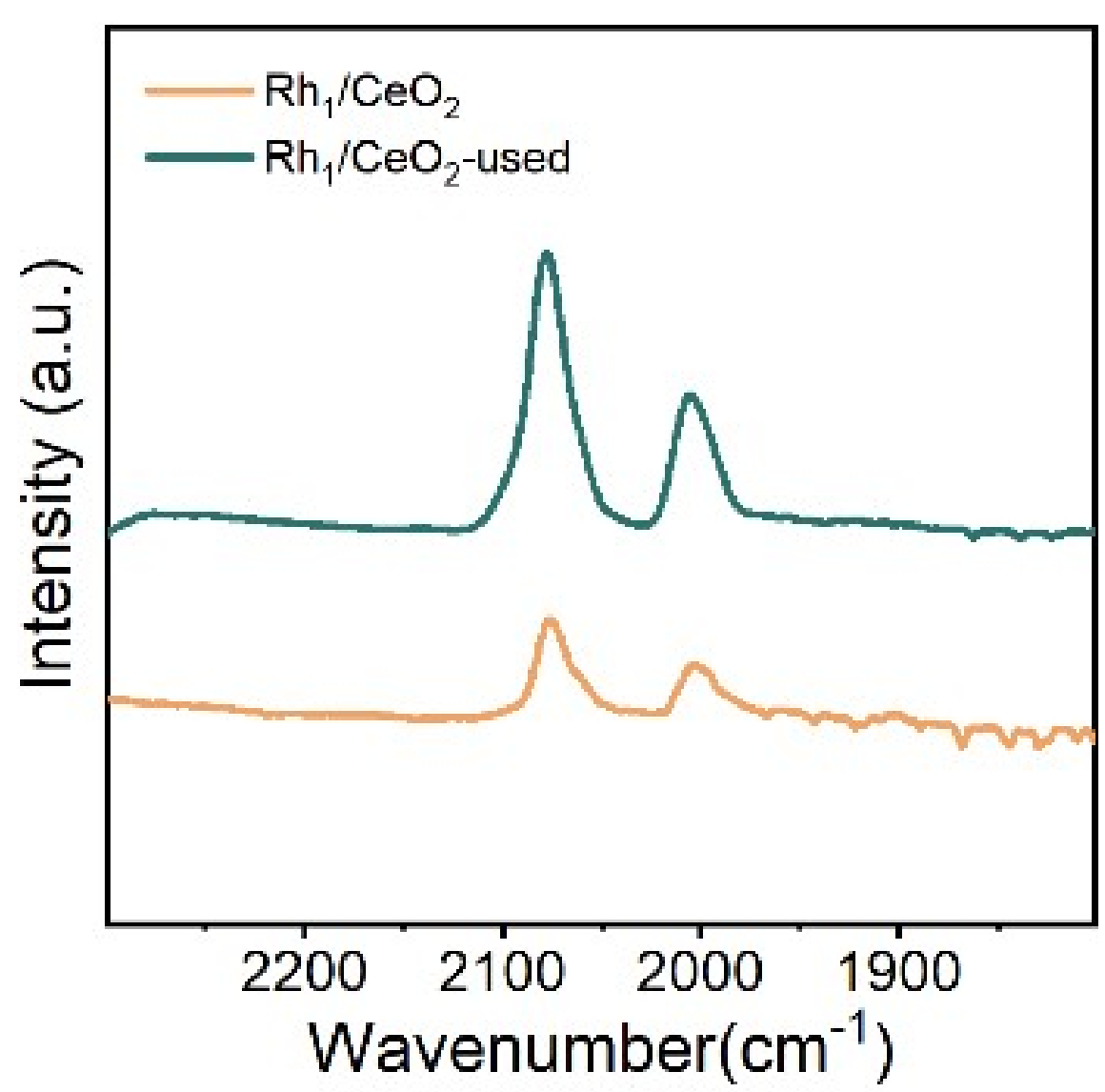

Figure S11. CO-FTIR curves for Rh_1/CeO_2 before and after reaction.

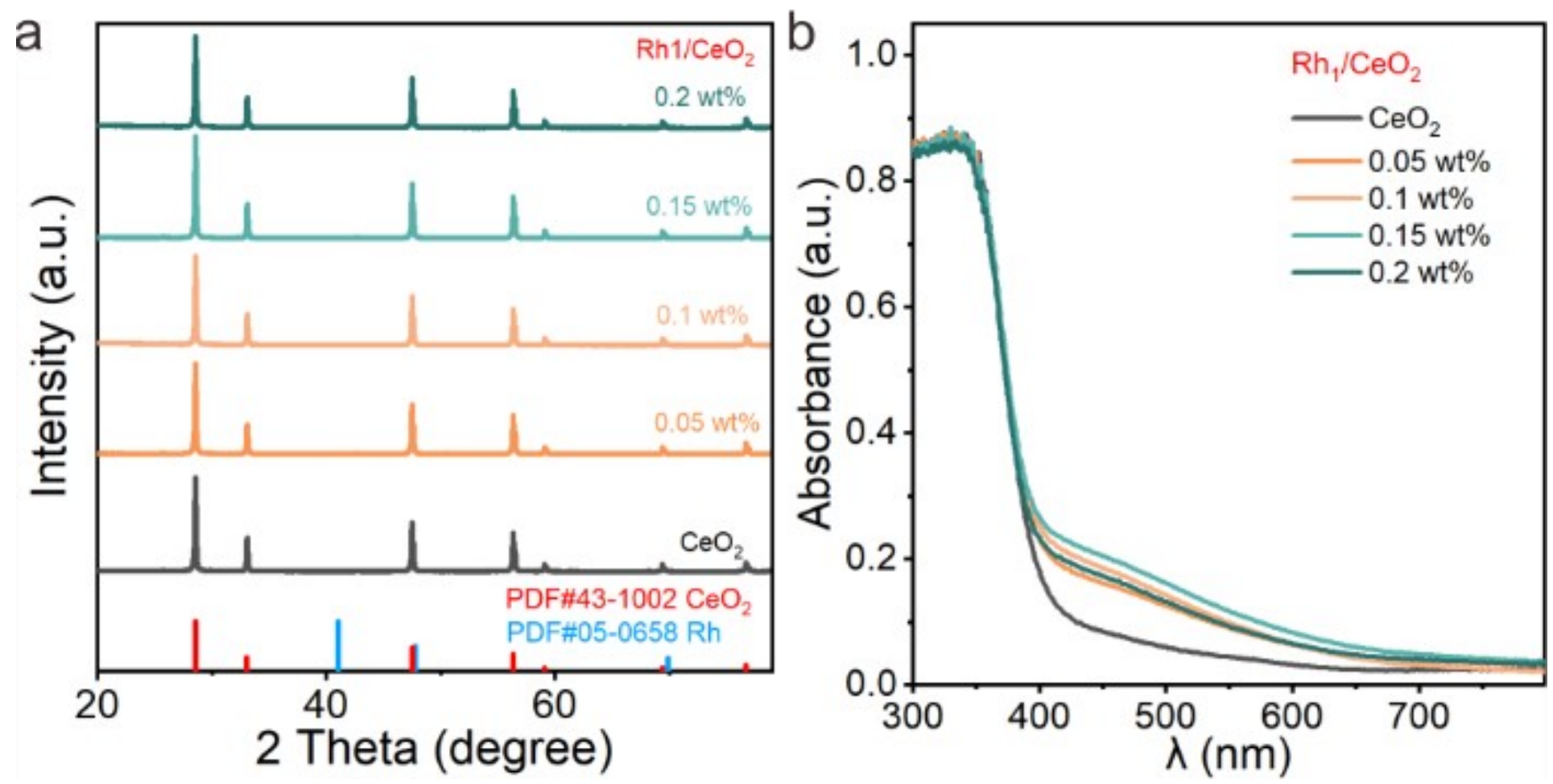

Figure S12. (a) XRD patterns and (b) UV-vis DRS of Rh_1/CeO_2 loaded with different content of Rh (0.05, 0.1, 0.15 and 0.2 wt%).

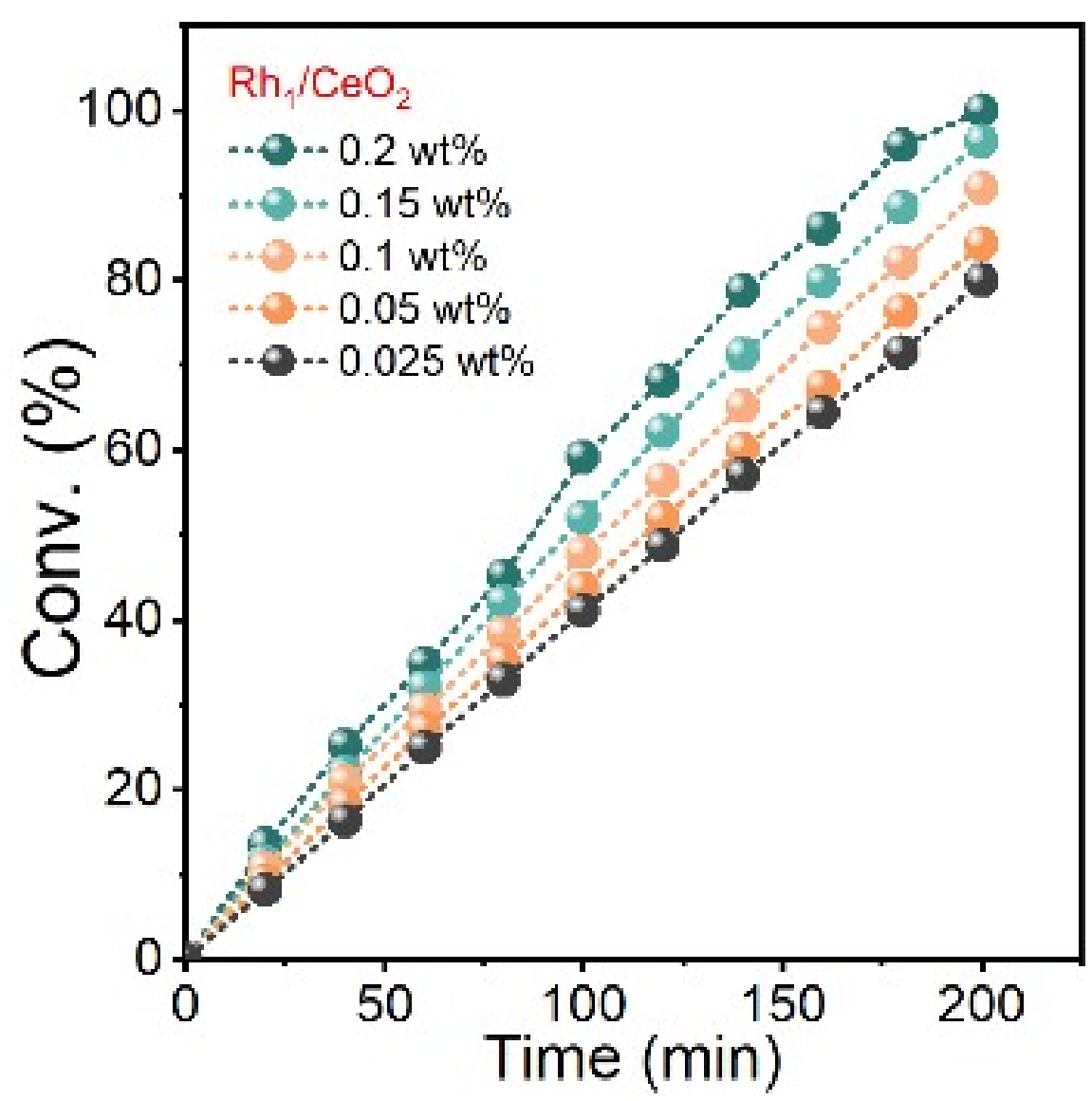

Figure S13. Conversion vs. time graphs of Rh_1/CeO_2 Rh_1/CeO_2 loaded with different content of Rh (0.05, 0.1, 0.15 and 0.2 wt%) with Xenon lamp light (λ> 300 nm) under 0.1MPa H_2.

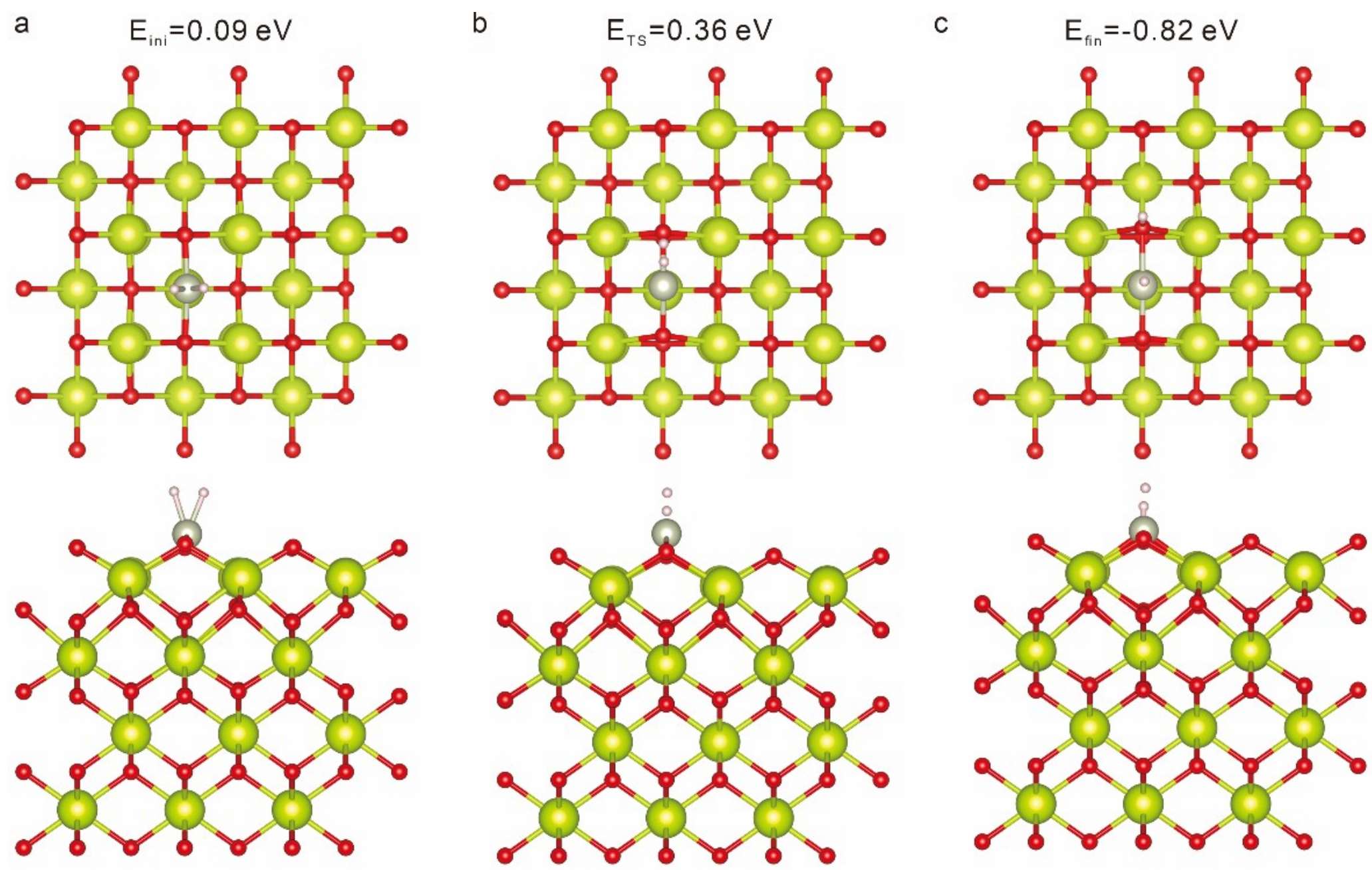

Figure S14. The top-view (above) and front-view (under) of (a) the initial state of H_2 activation at Rh_1/CeO_2 surface. (b) TS. (c) the final state.

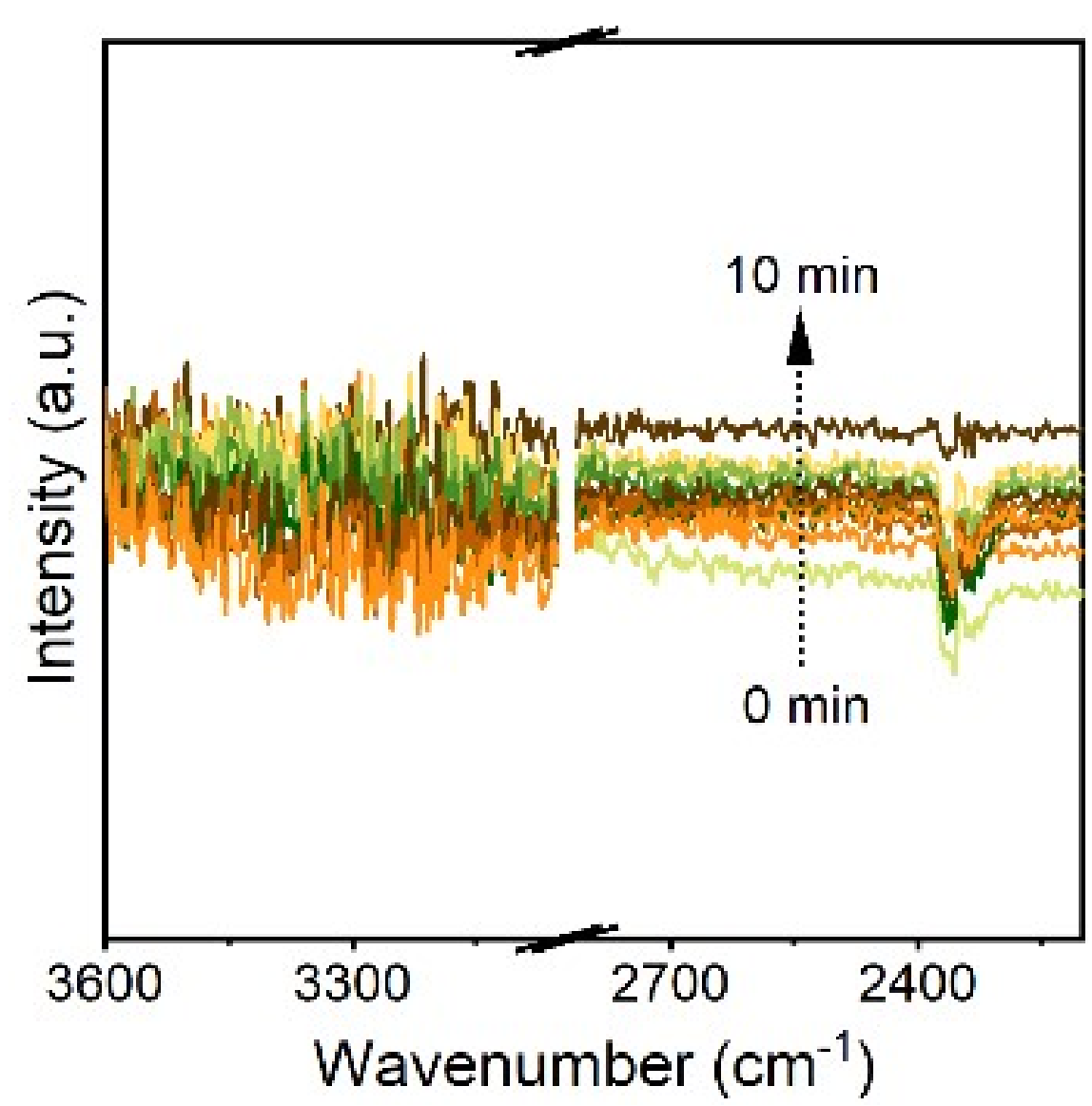

Figure S15. *in-situ* D_2-FTIR curves for CeO_2.

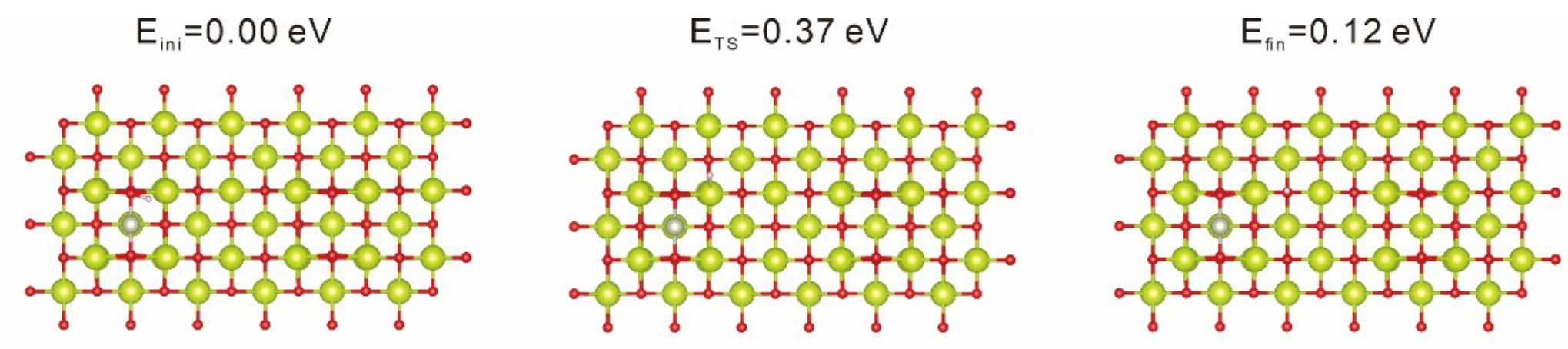

Figure S16. The top-view of the initial state, TS and the final state of H_2 transfer path from S1 to S2 at Rh_1/CeO_2 surface.

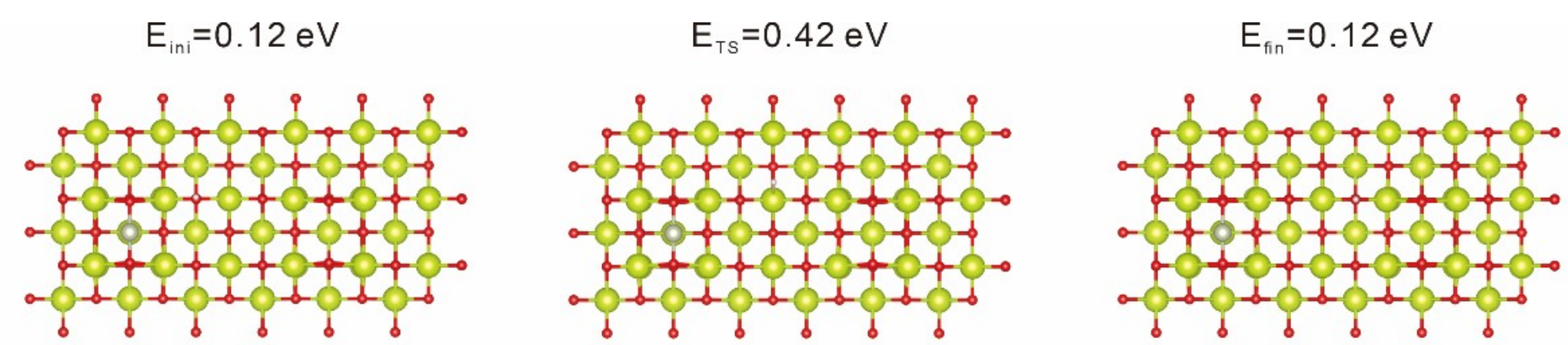

Figure S17. The top-view of the initial state, TS and the final state of H_2 transfer path from S2 to S3 at Rh_1/CeO_2 surface.

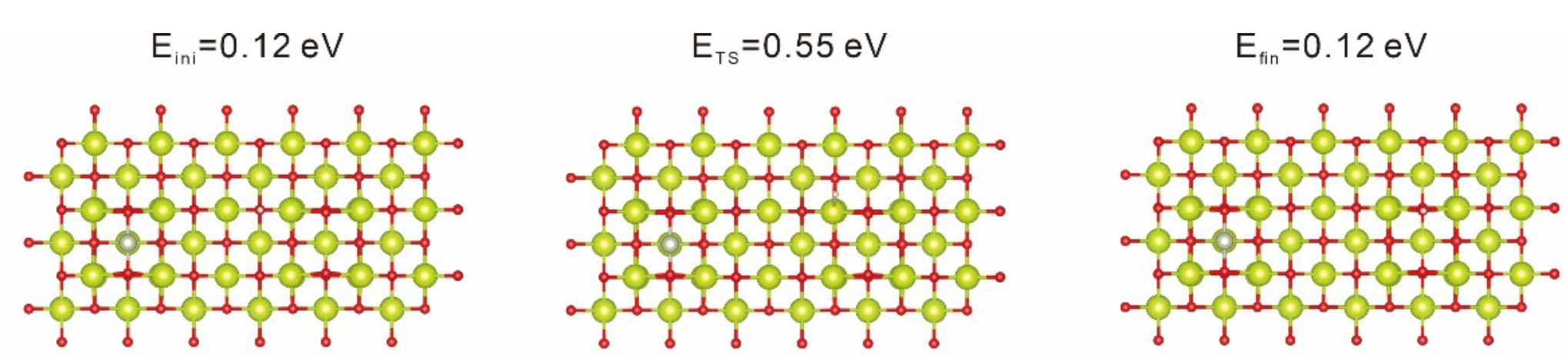

Figure S18. The top-view of the initial state, TS and the final state of H_2 transfer path from S3 to S4 at Rh_1/CeO_2 surface.

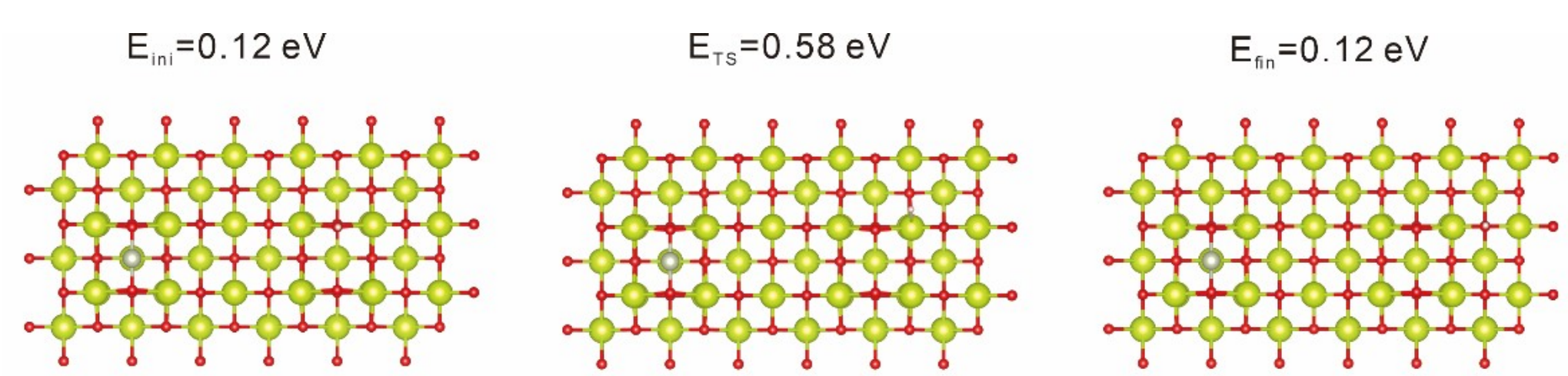

Figure S19. The top-view of the initial state, TS and the final state of H_2 transfer path from

S4 to S5 at Rh_1/CeO_2 surface.

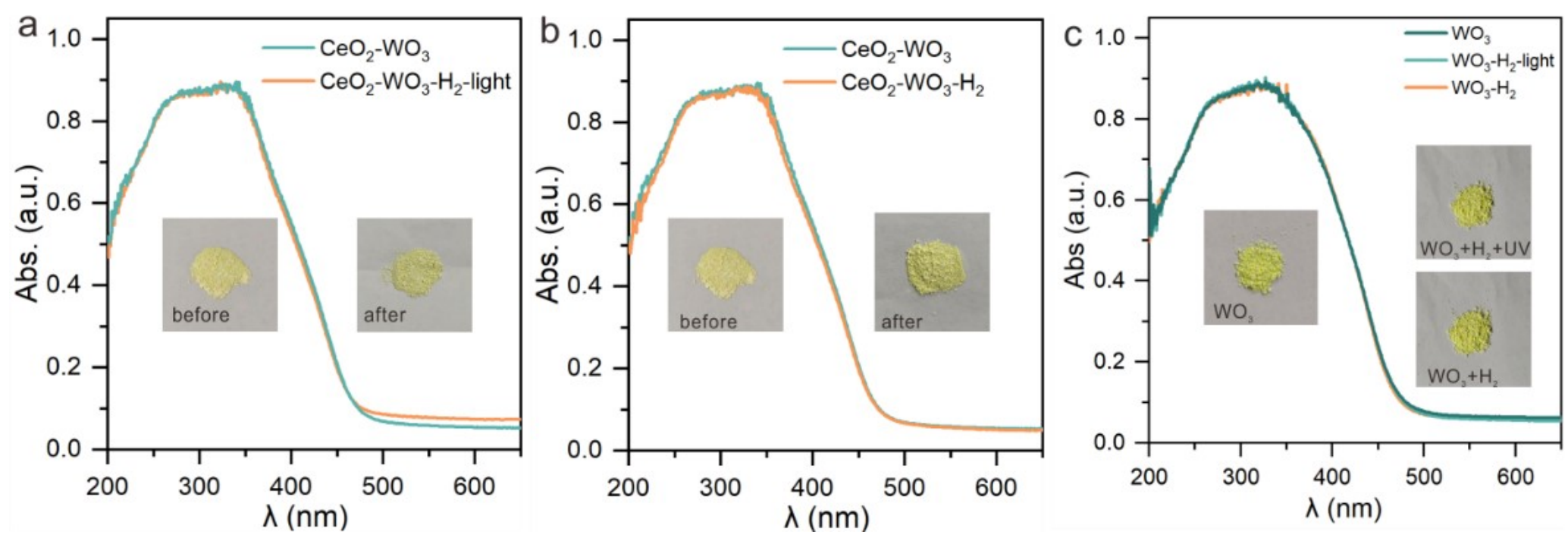

Figure S20. UV-vis DRS and pictures of (a) CeO_2 with WO_3 before and after dealt with H_2 and UV light. (b) CeO_2 with WO_3 before and after dealt with H_2. (c) WO_3 before and after dealt with H_2 and UV light.

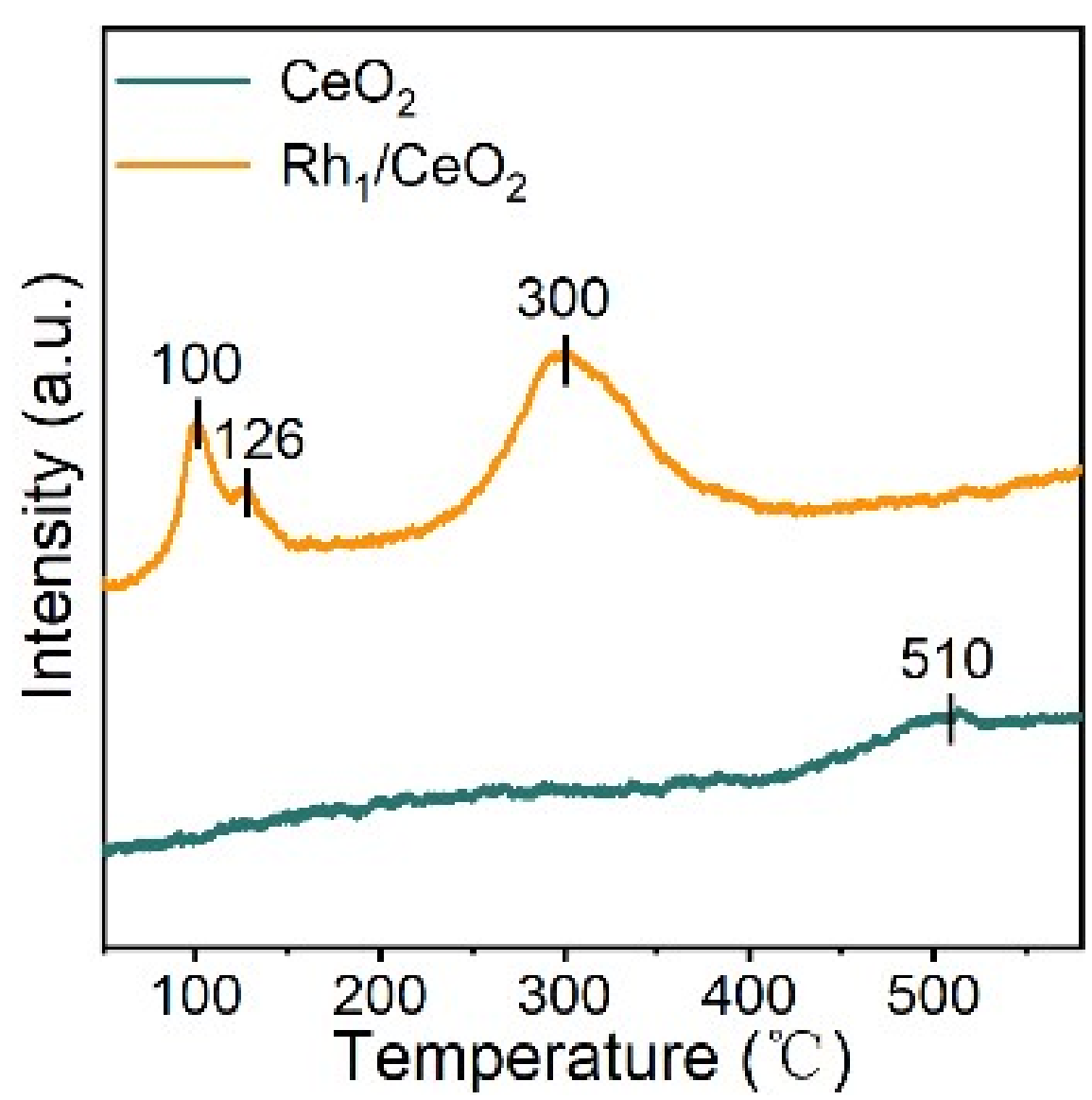

Figure S21. H_2-TPR of CeO_2 and Rh_1/CeO_2.

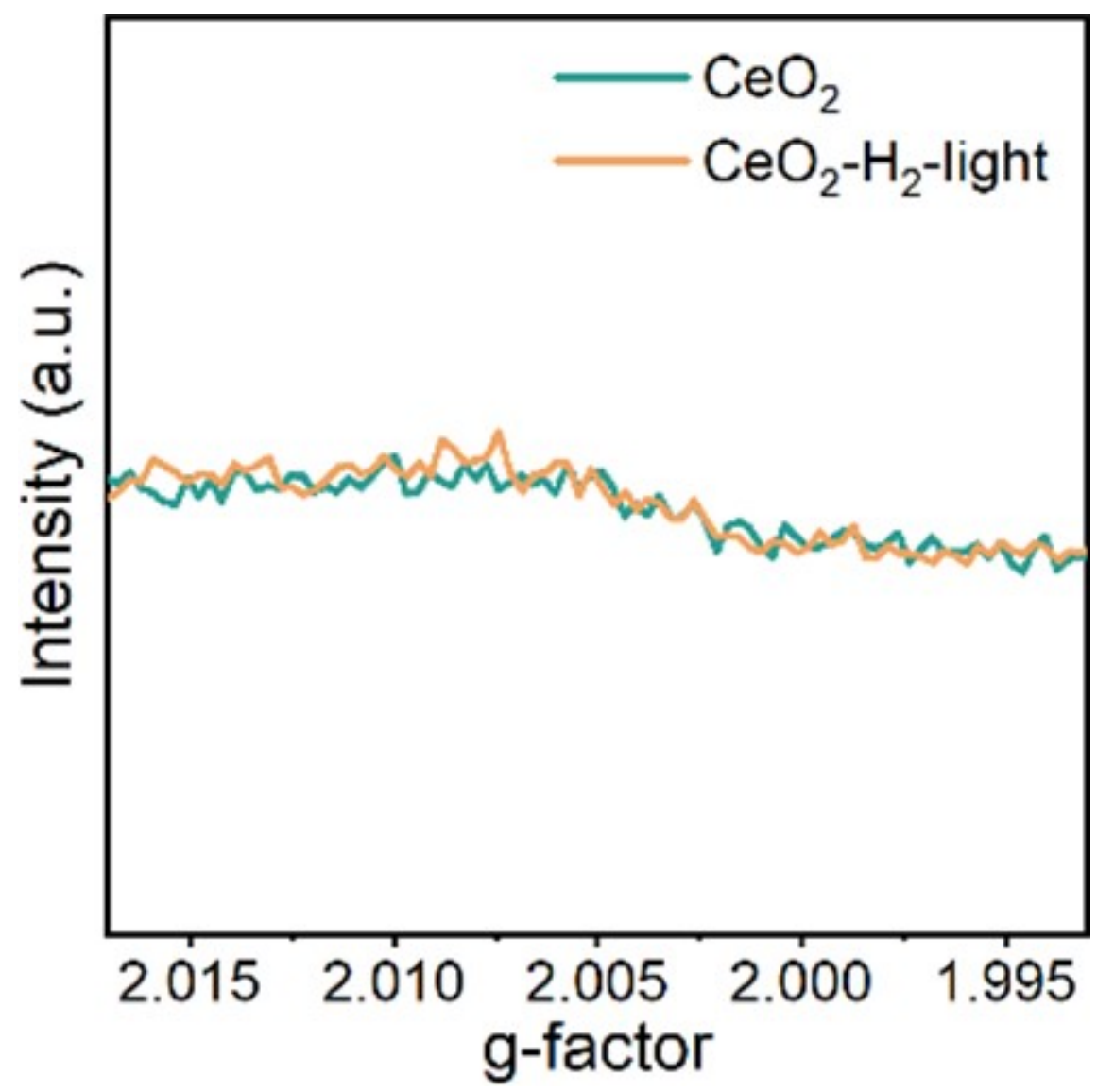

Figure S22. *in-situ* EPR curves of CeO_2 before and after UV irradiation.

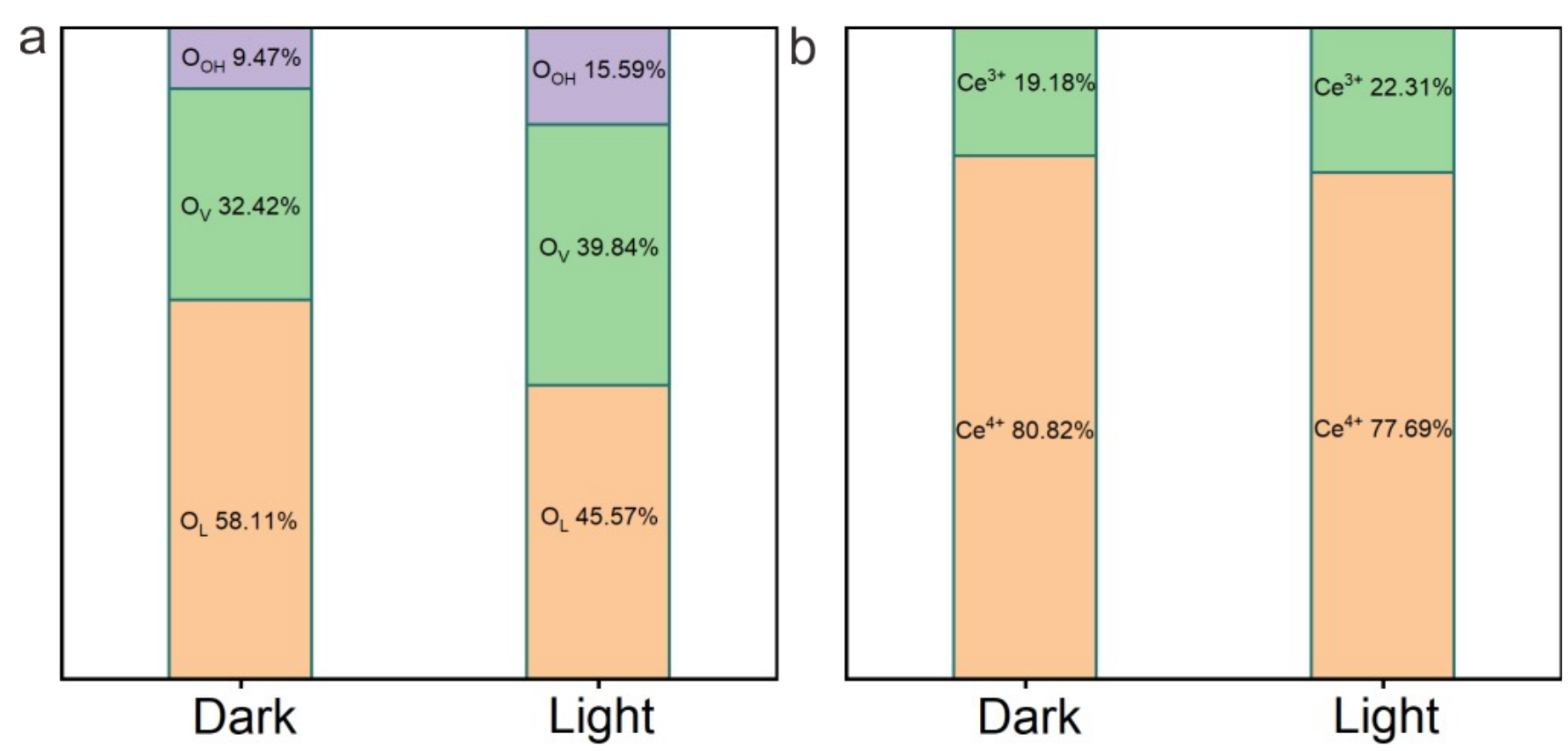

Figure S23. The proportion of each component of *in-situ* XPS spectra of Rh_1/CeO_2 before and after UV irradiation. (a) O 1s and (b) CeO_2 3d region.

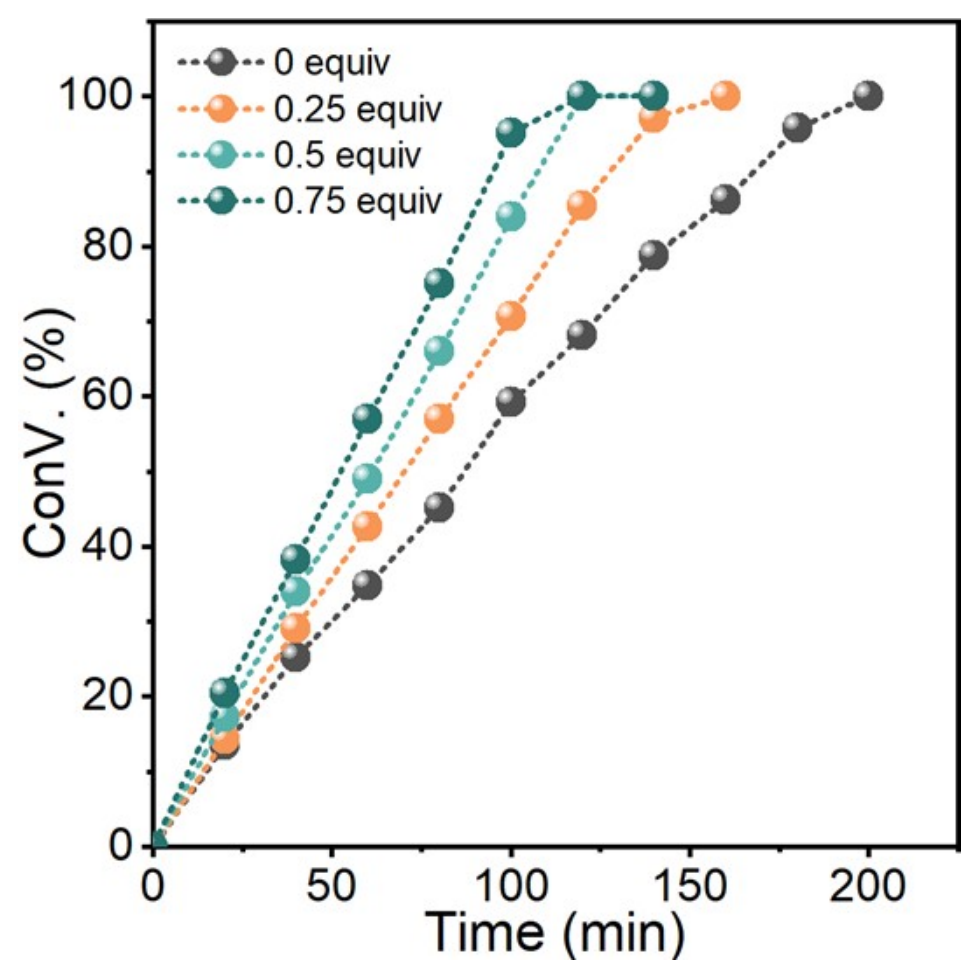

Figure S24. Conversion vs. time graphs of Rh_1/CeO_2 with different equiv. of CeO_2.

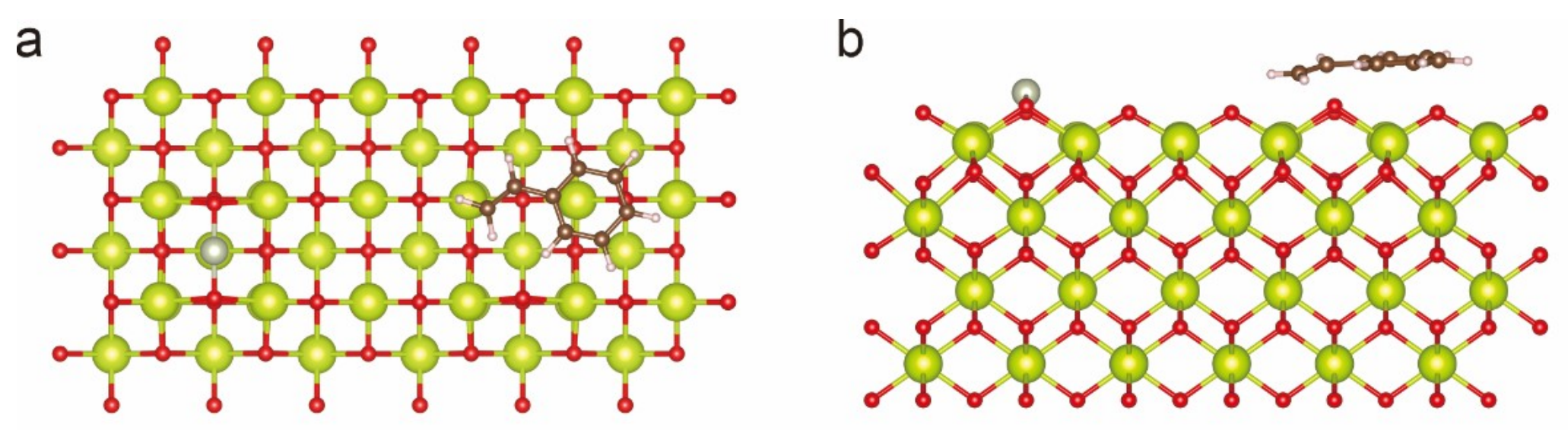

Figure S25. (a) The top-view and (b) front-view of theoretical model of Rh_1/CeO_2 adsorbed by styrene.

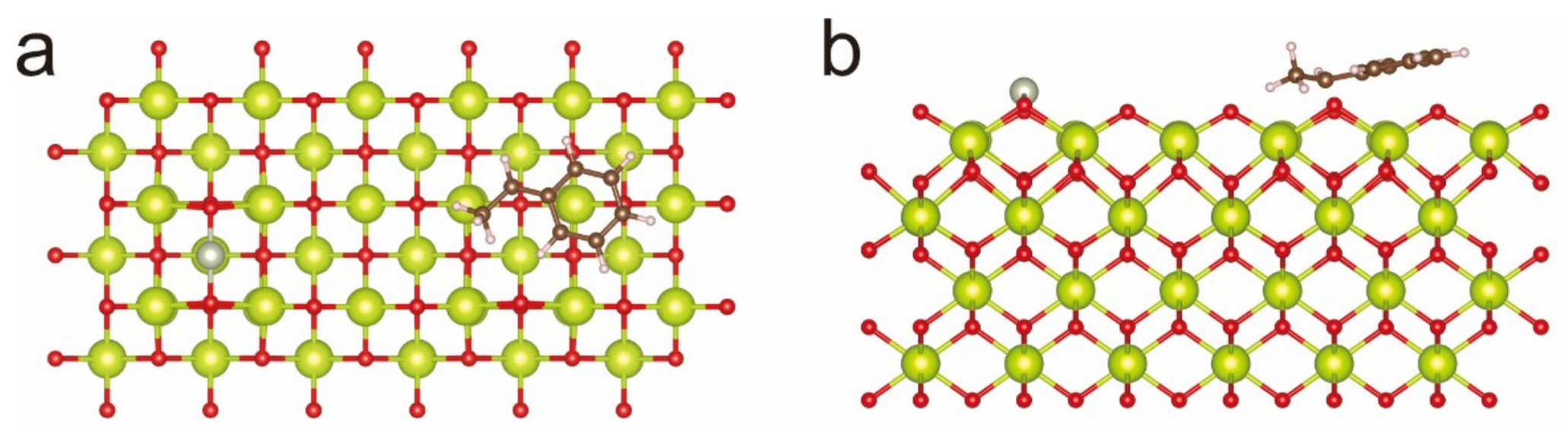

Figure S26. (a) The top-view and (b) front-view of theoretical model of reaction barriers for C_8H_8 to C_8H_9 on Rh_1/CeO_2 surface

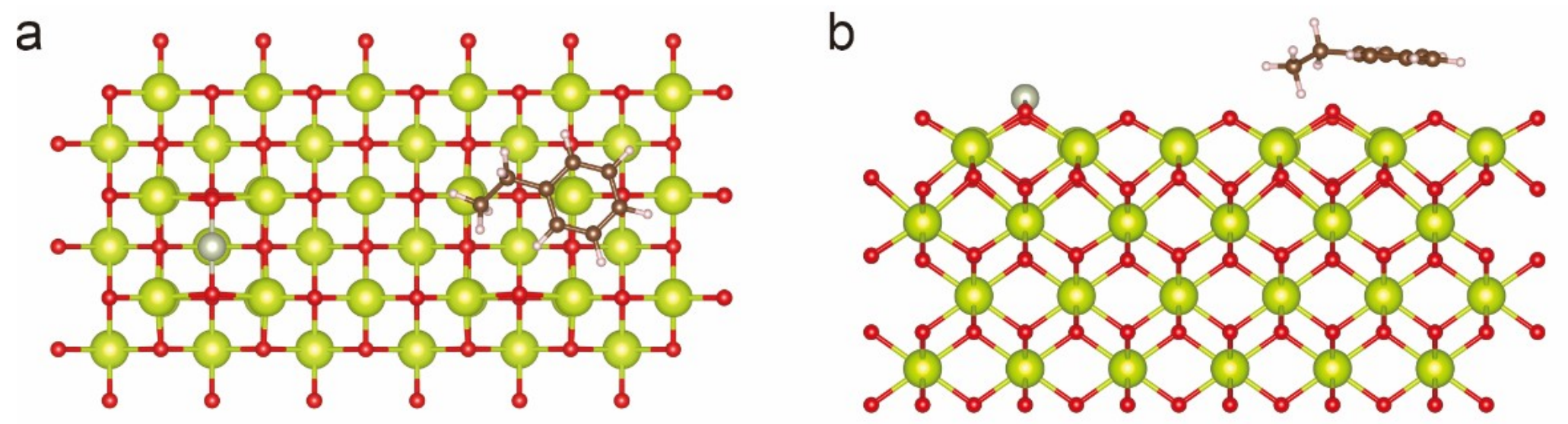

Figure S27. (a) The top-view and (b) front-view of theoretical model of reaction barriers for C_8H_9 to C_8H_{10} on Rh_1/CeO_2 surface.

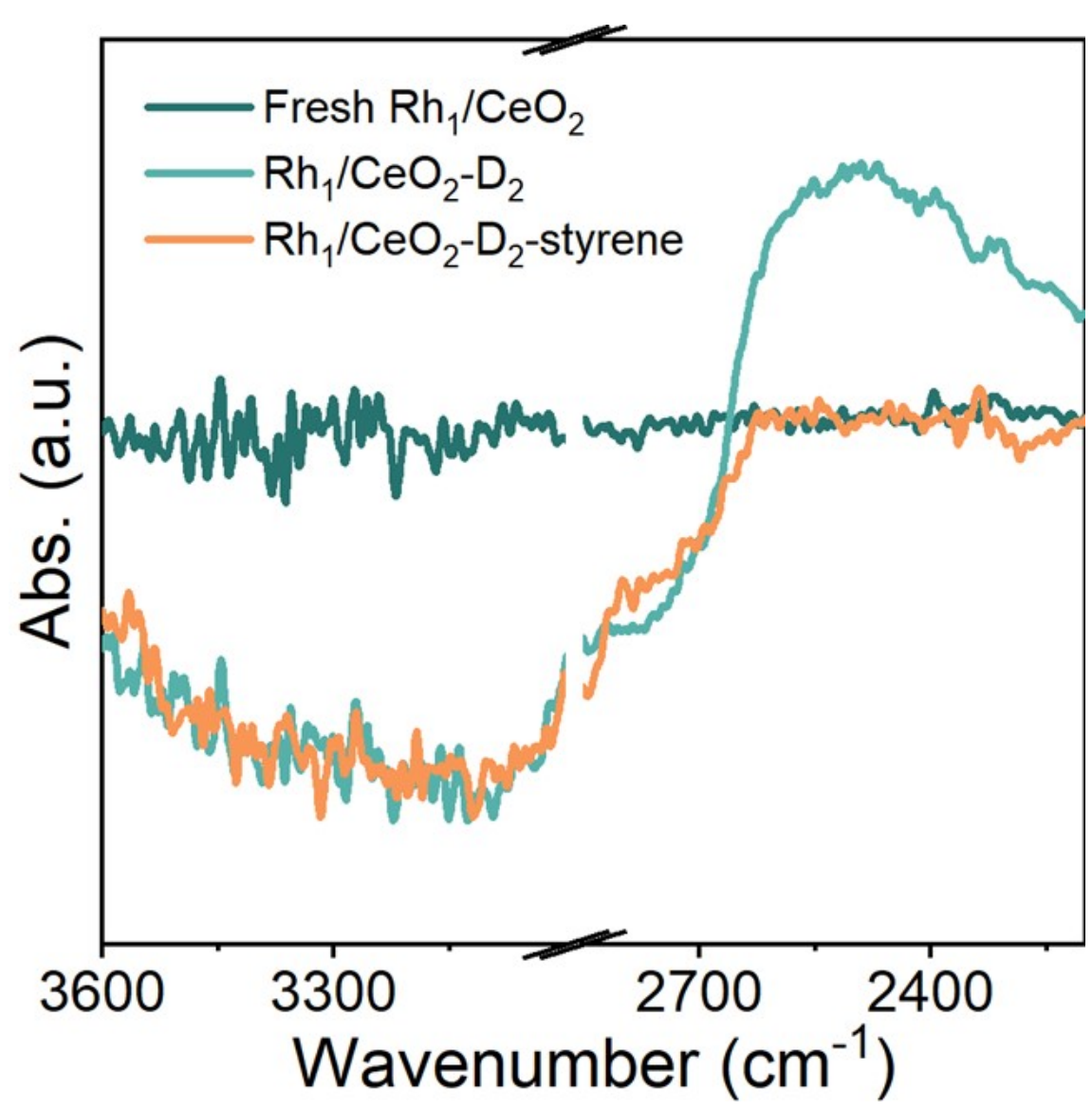

Figure S28. *In situ* FT-IR curves for Rh_1/CeO_2 by using styrene probe molecules under light.

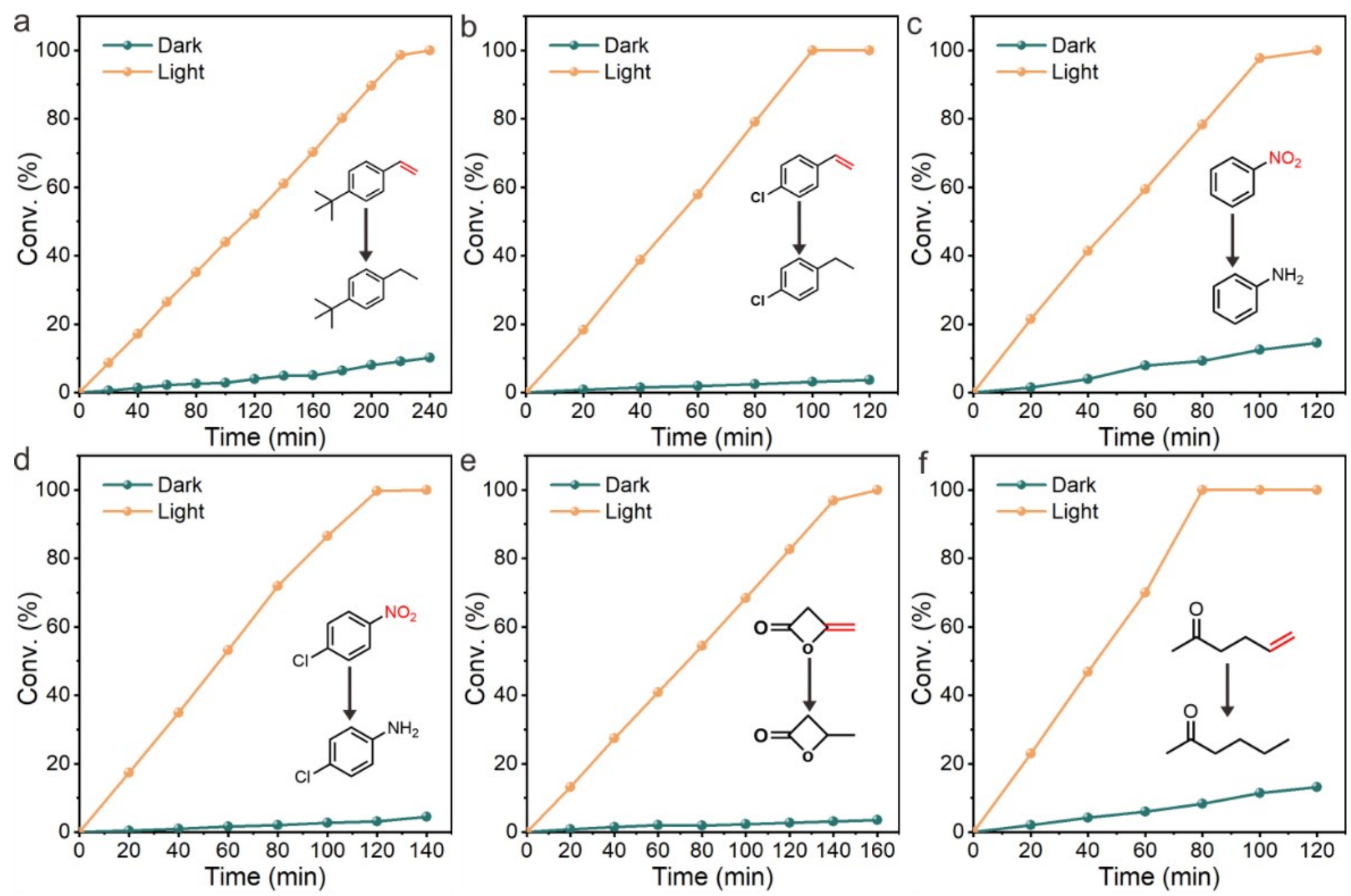

Figure S29. Conversion vs. time graphs of photocatalytic other unsaturated compounds hydrogenation over Rh_1/CeO_2: (a) 4-tert-butylstyrene, (b) 4-chlorostyrene, (c) nitrobenzene, (d) 4-chloronitrobenzene, (e) diketene, and (f) allylacetone.

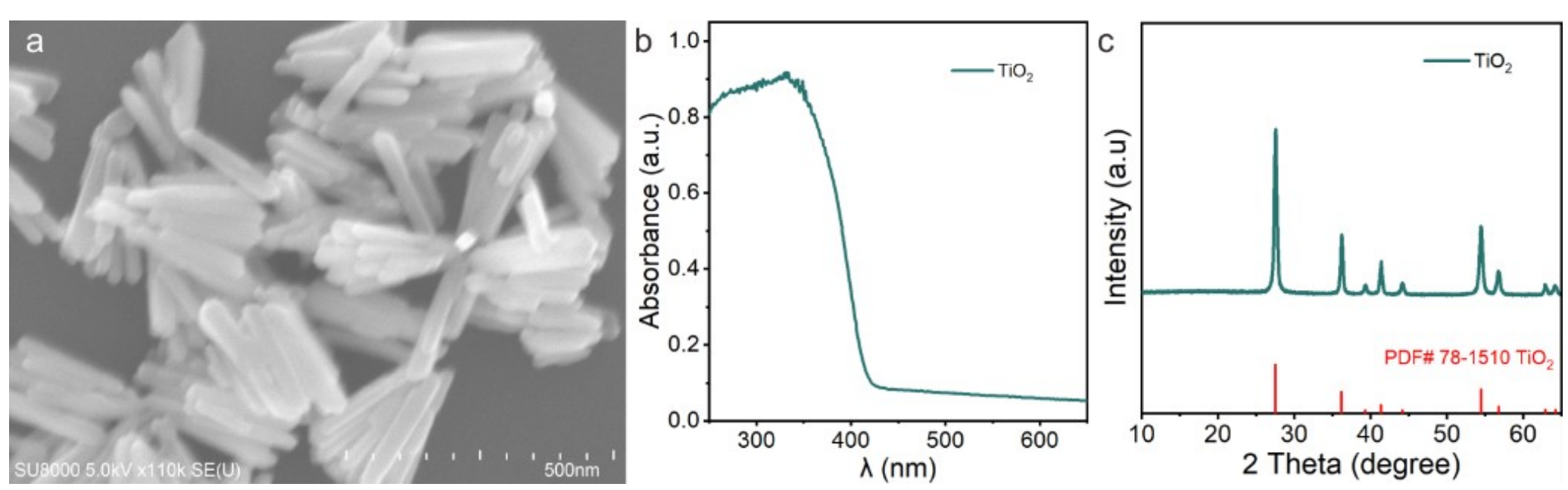

Figure S30. (a) SEM image, (c) UV-vis DRS and (b) XRD patterns of TiO_2.

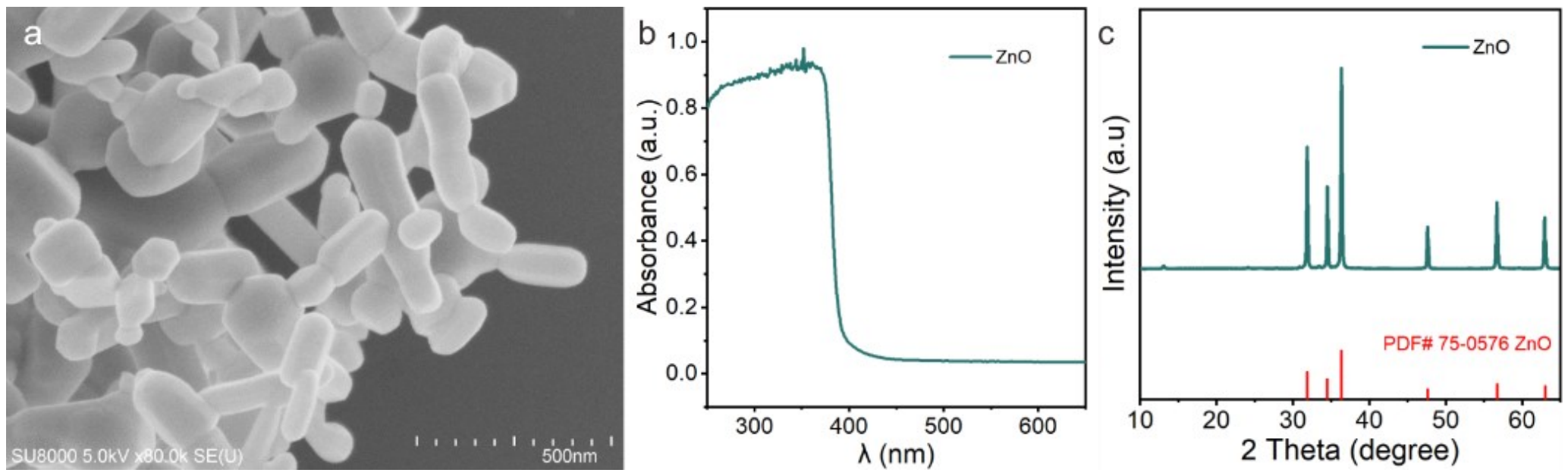

Figure S31. (a) SEM image, (c) UV-vis DRS and (b) XRD patterns of ZnO.

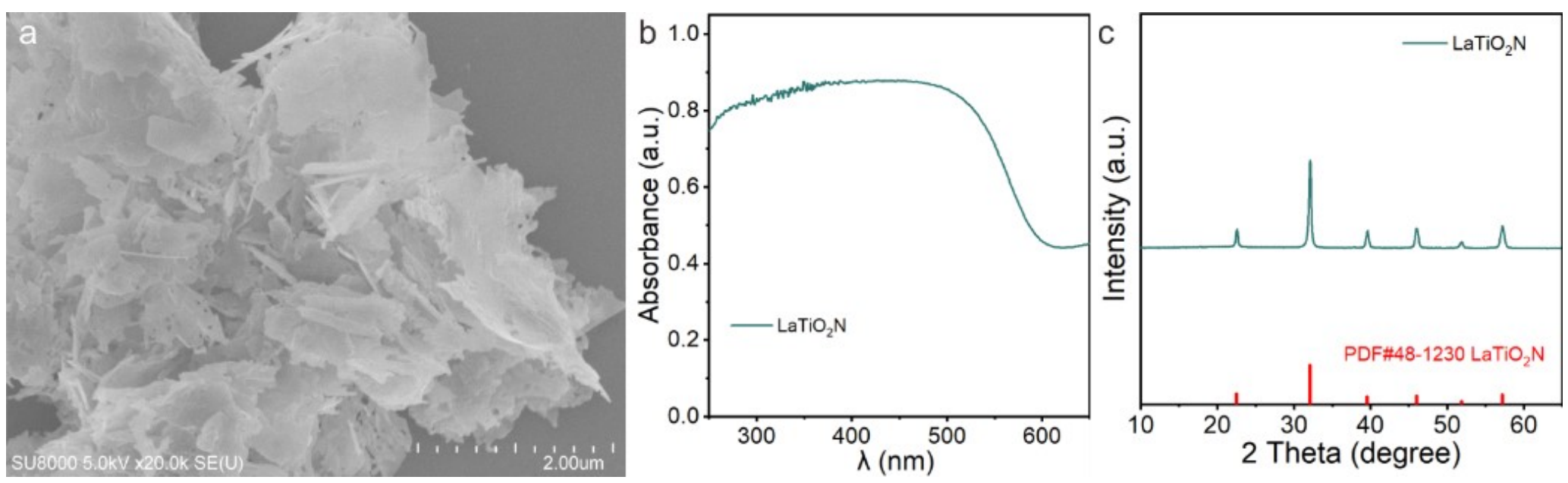

Figure S32. (a) SEM image, (c) UV-vis DRS and (b) XRD patterns of $LaTiO_2N$.

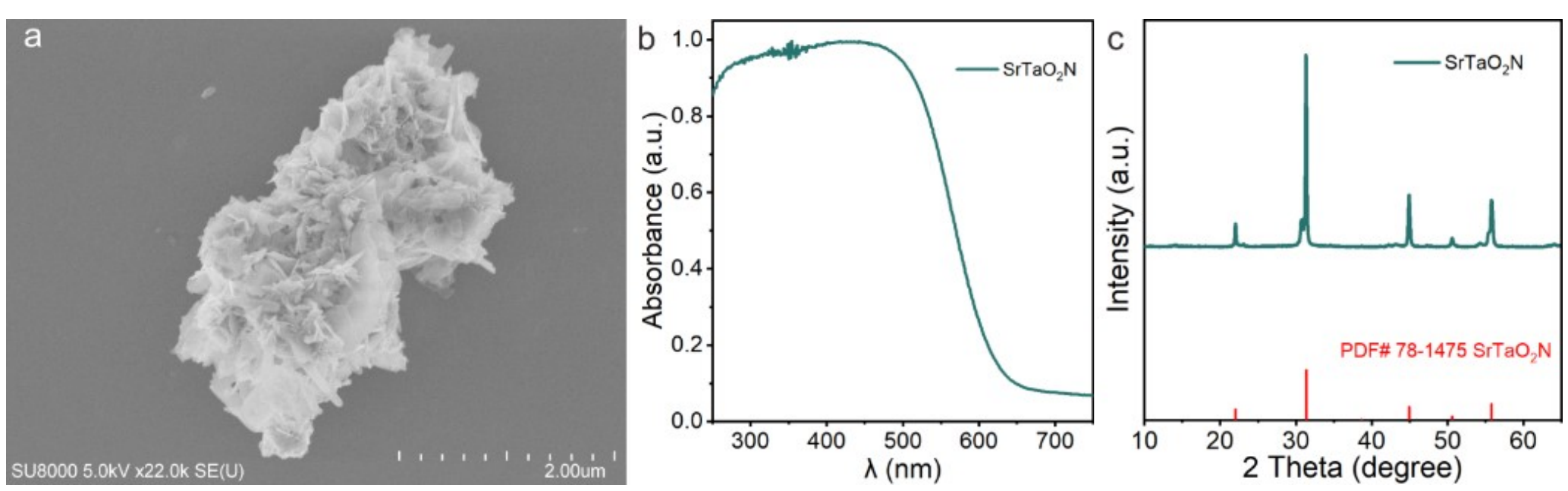

Figure S33. (a) SEM image, (c) UV-vis DRS and (b) XRD patterns of $SrTaO_2N$.

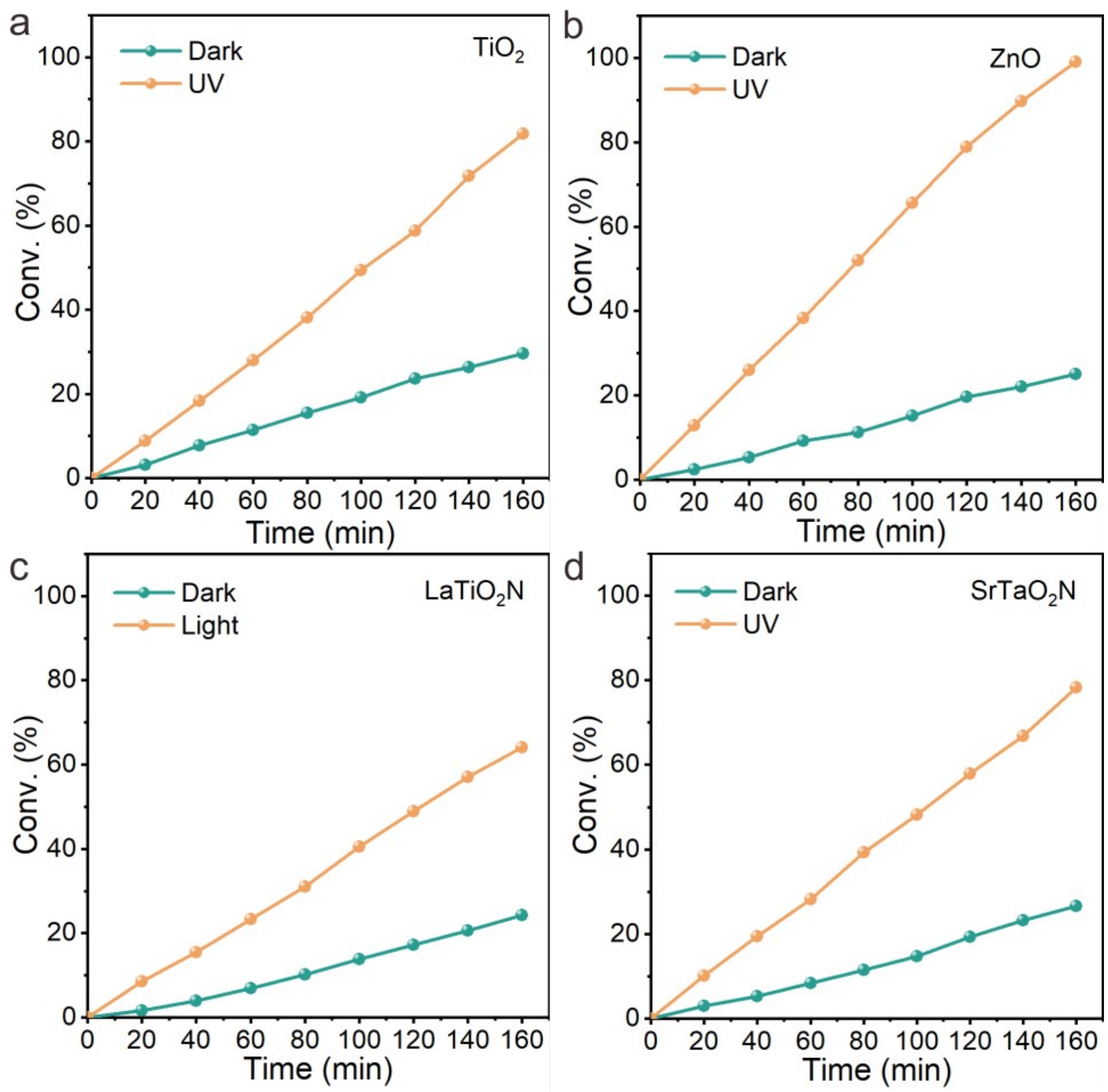

Figure S34. Conversion vs. time graphs of (a) TiO_2, (b) ZnO, (c) $LaTiO_2N$ and (d) $SrTaO_2N$.

Table S1. EXAFS Fitting Results for Rh foil, Rh_2O_3 and Rh_1/CeO_2 Catalysts.

**Sample**	**shell**	**CN**	**R (Å)**	**ΔE (eV)**	**σ^2 (Å^2)**	**R-factor**
Rh foil	Rh-Rh	7.5±0.3	2.48±0.02	2.8±0.9	0.002	0.0015
Rh_2O_3	Rh-O	3.9±0.7	2.04±0.07	0.2±2.16	0.00036	0.016
	Rh-O-Rh	0.9±0.6	3.11±0.02			
Rh_1/CeO_2	Rh-O	4.0±0.6	2.03±0.08	1.3±0.6	0.0018	0.0013

Table S2. The Rh content (wt%) of Rh_1/CeO_2[a]

Entry	Theoretical	Measured
1	0.2	0.191
2	0.15	0.143
3	0.10	0.099
4	0.05	0.048
5	0.025	0.023

[a] Determined by ICP-OES analysis.

References

1. G. Kresse and J. Furthmuller, Physical Review: B, Condensed Matter and Materials Physics, 1996, 54, 11169-11186.
2. G. Kresse and D. Joubert, Physical Review: B, Condensed Matter and Materials Physics, 1999, 59, 1758-1775.
3. P. E. Blochl, Physical Review: B, Condensed Matter and Materials Physics, 1994, 50, 17953-17979.
4. J. P. Perdew, K. Burke and M. Ernzerhof, Physical Review Letters, 1996, 77, 3865-3868.
5. H. J. Monkhorst and J. D. Pack, Physical Review B 1976, 13, 5188-5192.
6. S. Grimme, Journal of Computational Chemistry 2006, 27, 1787-1799.
7. G. Henkelman, B. P. Uberuaga and H. Jónsson, Journal of Chemical Physics, 2000, 113, 9901-9904.
8. A. A. Peterson, F. Abild-Pedersen, F. Studt, J. Rossmeisl and J. K. Nørskov, Energy & Environmental Science, 2010, 3, 1311-1315.
